# Sodium-Glucose Cotransporter Inhibitors as Antidiabetic Drugs: Current Development and Future Perspectives

**DOI:** 10.1021/acs.jmedchem.2c00867

**Published:** 2022-08-04

**Authors:** Rosanna Maccari, Rosaria Ottanà

**Affiliations:** Department of Chemical, Biological, Pharmaceutical and Environmental Sciences, University of Messina, Viale F. Stagno D’Alcontres, 31, 98166 Messina, Italy

## Abstract

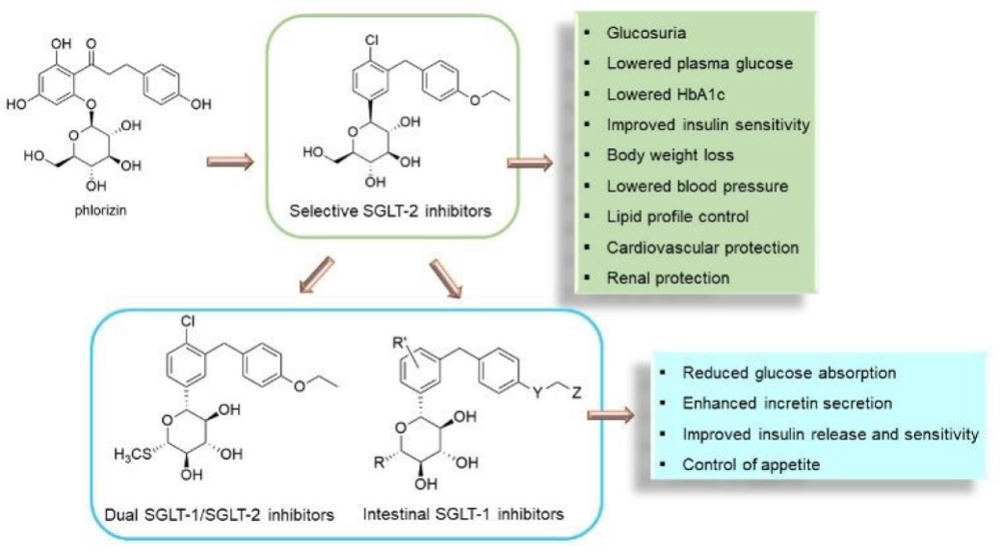

Sodium-glucose cotransporter 2 (SGLT-2) inhibitors (gliflozins) represent the most recently approved class of oral antidiabetic drugs. SGLT-2 overexpression in diabetic patients contributes significantly to hyperglycemia and related complications. Therefore, SGLT-2 became a highly interesting therapeutic target, culminating in the approval for clinical use of dapagliflozin and analogues in the past decade. Gliflozins improve glycemic control through a novel insulin-independent mechanism of action and, moreover, exhibit significant cardiorenal protective effects in both diabetic and nondiabetic subjects. Therefore, gliflozins have received increasing attention, prompting extensive structure–activity relationship studies and optimization approaches. The discovery that intestinal SGLT-1 inhibition can provide a novel opportunity to control hyperglycemia, through a multifactorial mechanism, recently encouraged the design of low adsorbable inhibitors selectively directed to the intestinal SGLT-1 subtype as well as of dual SGLT-1/SGLT-2 inhibitors, representing a compelling strategy to identify new antidiabetic drug candidates.

## 1 Introduction

Diabetes mellitus (DM) is a chronic complex disease, typically associated with a state of hyperglycemia which occurs as a result of scarce tissue responsiveness to insulin signaling (a condition known as insulin resistance) or insufficient secretion of the hormone by pancreatic β-cells. Hyperglycemia-related dysfunctions can cause tissue and vascular damage and, consequently, elicit the development of serious complications, such as nephropathy and cardiovascular diseases, which can cause disabling effects and threaten life for diabetic patients. In the past few years, the prevalence of DM has dramatically increased and turned out to be worse than expected. Currently, 537 million adults (over 10.5% of the adult population) are suffering from DM worldwide; this number is predicted to rise to 643 million by 2030 and 783 million (most likely over 12%) by 2045. Moreover, DM ranks among the top causes of premature death and was responsible for 6.7 million deaths in 2021. It is also estimated that 541 million adults suffer from impaired glucose tolerance (IGT), a condition which places them at high risk to develop type 2 diabetes (T2DM).^1,2^ More than 90% of diabetic patients suffer from this latter type of DM, which is mainly related to the resistance of the target organs to insulin and, over time, also to a progressive reduction in the ability of β cells to produce insulin, with a partial deficiency in the amount of available hormone. The other main type of DM is type 1 diabetes (T1DM), characterized by the autoimmune destruction of pancreatic β cells, which generally culminates in the total inability to secrete insulin.

Although several therapeutic options are available for the treatment of DM, the current therapy can only slow the progression of this disease, essentially by controlling blood glucose levels to prevent dysfunctions and complications caused by hyperglycemia. In addition, the clinical use of certain available antidiabetic drugs can be accompanied by the occurrence of undesired side effects, such as body weight gain and hypoglycemia, or can show limited efficacy in regulating glycemic homeostasis in some patients. Therefore, the necessity of safer and more effective therapeutic strategies requires continuous research efforts in this field, thus allowing a number of antidiabetic drugs directed to different biological targets to be obtained. One of the advantages of having antidiabetic agents endowed with different mechanisms of action lies in the opportunity of using them in combination therapy to achieve more effective control of the different metabolic and tissue dysfunctions implicated in the progression of DM.

Inhibitors of the renal sodium-glucose cotransporter 2 (SGLT-2) represent the most recently approved class of orally active antidiabetic drugs. SGLT-2 is a protein mainly expressed in the kidneys, where it is responsible for the tubular reabsorption of most of the filtered glucose. Since it plays a major role in the renal glucose reabsorption, SGLT-2 was assumed as a therapeutic target of considerable interest, culminating in 2012 in the approval of dapagliflozin, the first antidiabetic drug active as an SGLT-2 inhibitor, followed by several analogues that entered clinical use in the past decade. In diabetic animal models, highly increased expression of renal SGLT-2 was observed compared with normal controls; moreover, in proximal tubular cells isolated from T2DM patients, both SGLT-2 levels and glucose reabsorption were found to be considerably higher than in controls, suggesting that a possible hyperglycemia-induced overexpression of glucose transporters contributes to exacerbate the hyperglycemic condition typical of DM.^3,4^

Despite concerns about certain side effects of SGLT-2 inhibitors, such as increased incidence of genitourinary infections, ketoacidosis, and bone fractures,^5^ these oral antidiabetic drugs were welcomed due to their novel insulin-independent mechanism of action and received increasing attention, thus prompting the development of new derivatives. Compelling evidence demonstrated that SGLT-2 inhibitors not only are able to lower both blood glucose and glycated hemoglobin (HbA1c) levels but can also control body weight, blood pressure, lipidemic profiles, and endothelial functions and improve the efficiency of cardiac output. These actions result in important renal- and cardioprotective effects, which can reduce the incidence of serious cardiovascular complications often associated with DM.^6−12^

Later, it was demonstrated that inhibition of SGLT-1 cotransporter, which is mainly expressed in the intestine, can also provide a novel important contribution to glycemic control by means of multifactorial mechanisms, resulting in both significant reduction of intestinal glucose absorption and increased release of incretins by the enteroendocrine cells; these latter hormones play decisive roles in improving cellular response to insulin signaling, ameliorating β cell functionality, and exerting cardio- and neuroprotective effects, thus providing a fundamental contribution to the overall glycemic control. These findings further motivated the design and evaluation of new SGLT inhibitors, with the aim of identifying novel antidiabetic candidates.^13−15^

The development of SGLT inhibitors was mainly based on extensive structure–activity relationship (SAR) studies, whereas the knowledge of the target structures has been so far insufficient to support the structure-based design of new inhibitors. In this review, we report the main steps of the development of SGLT inhibitors, highlighting SARs, recent advancements, and prospects of this class of antidiabetic drugs and discussing them from a medicinal chemistry point of view.

## 2 Sodium-Glucose Cotransporters SGLT-1/SGLT-2: Attractive Molecular Targets for Drug Development

SGLT proteins belong to the SLC5 solute carrier family, in turn included in the wider family of sodium-solute symporters, which comprises carriers present in most living beings, capable of mediating the transport of a number of small organic molecules, such as sugars, vitamins, and amino acids.^16^ The first member of the family to be cloned was the intestinal sodium-glucose cotransporter SGLT-1; numerous (more than 250) other proteins of the family were subsequently identified in cells of different species.^17−19^ Among them, the X-ray solved structure of the sodium-galactose symporter of *Vibrio parahemolyticus* (vSGLT) was taken as a model, due to structural and functional similarities with other members of the family and also with the Na^+^-leucine cotransporter included in the neurotransmitter-sodium symporter family.^18,20^ The vSGLT symporter has 14 transmembrane helices, with a core consisting of two inverted series of five transmembrane helices, which turned out to be a common structural aspect of other SGLT proteins. Galactose is bound in the center of this motif, about halfway across the membrane bilayer, and is surrounded by hydrophobic residues from TM1, TM2, TM6, TM7, and TM10 helices that can function as intracellular and extracellular gates.^18^ Molecular dynamics and biochemical studies suggested that Na^+^-sugar symport involves a conformational equilibrium between outward-facing and inward-facing protein conformations, through opening and closing of external and internal barriers composed by hydrophobic side-chains of inner transmembrane helices, such as TM2, TM6, and TM10. In particular, Na^+^ binding to its site in the outward-facing conformation is required to open an external gate and consequently allow the sugar to reach its substrate-binding site; then, the outer gate closes, blocking the sugar into the binding pocket, and subsequently the opening of an internal gate allows both Na^+^ and glucose to reach the cytoplasm. Finally, the inner gate closes and the protein isomerizes to its initial conformation.^16,18−20^ This cycle is reversible and depends on the external and internal concentrations of Na^+^ and glucose as well as on the membrane potential. In addition, it was observed that the conformational change induced by Na^+^ binding not only opens a large vestibule that accesses the sugar-binding site but also determines an increase of polarity in the pocket walls and enhances sugar affinity, thus suggesting an induced-fit mechanism for the binding of both substrates and inhibitors.^21,22^

Similarly to SGLT-1, SGLT-2 was cloned and structurally characterized more than two decades ago,^23^ but an exhaustive understanding of its transport mechanism has not been achieved yet; only recently it was revealed that SGLT-2 is coexpressed with MAP17, a small protein that interacts with the cotransporter and acts as an activator necessary to augment SGLT-2 symporter functions.^16^

Recently, starting from the X-ray solved structure of a *N*-acetylneuraminic acid transporter from *Proteus mirabilis* assumed as a template of the outward-facing conformations of both human SGLT-1 (hSGLT-1) and human SGLT-2 (hSGLT-2), a combined computational and functional study allowed interesting models of both hSGLT-1 and hSGLT-2 to be developed, thus providing structural insights into the possible binding modes of both substrates and inhibitors.^21^ Mutagenesis and computational data indicated that glycoside SGLT inhibitors can occupy both the sugar pocket and a predominantly hydrophobic outer vestibule. Upon inhibitor binding, partial closure of the outer gate could occur, through movements of TM9 and TM10 helices; these rearrangements induce the long flexible extracellular loop 5 (EL5c), which connects TM5 and TM6 helices, to cover the ligand, thus resulting in an induced-fit mechanism that leads to a partially occluded conformation of the symporter with both outer and inner gates closed.^21^ Moreover, docking experiments in both hSGLT-1 and hSGLT-2 outward-facing models suggested that the ligand binding mode is very similar between the two cotransporter subtypes, especially in the conserved glucose binding sites; many hydrophobic aromatic residues in the outer vestibule are also conserved, but sequence differences exist in the EL5c loops of the two transporters, which could be critically involved in subtype selectivity.^21^ Differences in Na^+^/glucose stoichiometry ratios between SGLT-1 and SGLT-2 (2/1 and 1/1, respectively) also were significant to explain differences in ligand affinity toward the two symporter subtypes. A putative SGLT-1 binding site for the second Na^+^ ion was proposed, which is characterized by the critical presence of Thr395, which is replaced by alanine in SGLT-2, thus preventing the binding of a second Na^+^ ion in this latter subtype. Since the binding of the second Na^+^ ion appeared to be related to the observed higher affinity of glucose for SGLT-1 over SGLT-2, it was suggested that the second Na^+^ ion could allosterically control the opening of the outer gate, thus favoring and stabilizing the open conformation that can bind glucose. On the other hand, the lack of a second Na^+^ ion binding site in SGLT-2 could favor the partially occluded conformation and inhibitor binding, thus determining, along with EL5c, significant differences in both substrate and inhibitor affinity toward the two symporter subtypes.^21^

Mutations in SGLT-encoding genes allowed a better understanding of the physiological roles played by these cotransporters, validating SGLT-2 as potential therapeutic target; in fact, initially, selectivity toward SGLT-2 over SGLT-1 was considered as a mandatory requirement for inhibitors as potential antidiabetic drugs, and only in a subsequent phase of the research it was demonstrated that partial SGLT-1 inhibition can lead to the identification of new drug candidates. In particular, hSGLT-1 deletion, due to rare mutations in the Na^+^-glucose cotransporter gene *SLC5A1A*, can cause a potentially lethal glucose-galactose malabsorption (GGM) disease in newborn individuals; these patients show little or no glucosuria, thus demonstrating that SGLT-1 is not the main responsible for renal glucose resorption. In addition, mutations in the *SLC5A2* gene, which encodes SGLT-2 subtype, cause a condition known as familial renal glycosuria, which is associated with urinary glucose excretion (UGE), polyuria, polydipsia, and increased urinary tract infections; however, most affected individuals do not exhibit relevant clinical symptoms or serious complications, such as hypoglycemia, and therefore this rare disease is considered benign.^16,24^

In healthy subjects, more than 99% of glomerular-filtered glucose (180 g per day) is reabsorbed in the renal proximal tubule. This glucose reabsorption process is mediated by SGLT cotransporters and glucose transporters (GLUT).^3,24^ The main factor responsible for the reabsorption of glucose from glomerular ultrafiltrate is hSGLT-2, whose expression is almost exclusively confined to the first tract (S1 and S2 segments) of the proximal renal tubule. In particular, SGLT-2 is mainly located in the luminal membrane of the tubular cells and acts as a high-capacity and low-affinity sodium-glucose cotransporter, which is responsible for the reabsorption of about 90–97% of filtered glucose. Renal glucose reabsorption also requires active removal of Na^+^, which is elicited by the electrochemical gradient continuously maintained by the Na^+^/K^+^ ATPase present in the basolateral membrane of the tubular cells and is responsible for Na^+^ current from cells inside to the plasma. In turn, the increased concentration of glucose within tubular cells activates the GLUT-2 carrier, which transports glucose through the basolateral membrane in the plasma direction, following the concentration gradient.^3,24^ In the distal section (S3 segment) of the renal proximal tubule, the remaining glucose amount (3–10% of the filtered glucose) is reabsorbed by SGLT-1, which acts as a low-capacity and high-affinity cotransporter. Differently from SGLT-2, the SGLT-1 subtype is present not only in the kidneys but also in other tissues and particularly in the intestine, where it is predominantly expressed and critically involved in the process of absorption of both glucose and galactose.^16^

Renal glucose reabsorption is a saturable process since in healthy subjects the renal tubule has a maximum capacity of glucose resorption (TmG) of about 375 mg/min. Under hyperglycemic conditions, the filtered glucose amount can exceed this threshold, and, consequently, glucose excretion in the urine (glucosuria) occurs. However, in T2DM patients, the mean renal threshold for glucose excretion (RT_G_), which is the plasma glucose concentration at which TmG is exceeded, is higher than in healthy subjects;^3,4,16^ this finding suggests that in DM adaptive mechanisms of the body aimed at avoiding the loss of energy source occur, which in turn could aggravate the hyperglycemic condition.

On the basis of its crucial role in renal glucose reabsorption, SGLT-2 emerged as a new molecular target for the development of antidiabetic drugs capable of acting through a novel mechanism of action; consequently, in the past decade, SGLT-2 inhibitors were developed as new therapeutic agents (gliflozins), which can appreciably improve the management of T2DM.^25−29^ In contrast to most antidiabetic agents, the anti-hyperglycemic effect resulting from SGLT-2 inhibition is totally insulin-independent, being related to the glucose amount filtered by the renal glomerulus that reaches the proximal tubule daily. The reduction of glycemic levels, in turn, leads to a lower percentage of protein glycation, enhanced insulin sensitivity of both liver and peripheral tissues, and improved functionality of insulin-producing pancreatic β cells, without inducing hypoglycemia. As a result of reduced hepatic insulin resistance, glucose production through hepatic gluconeogenesis, which is typically high in T2DM, can gradually decrease reaching normal values. In addition, the increased excretion of glucose through the kidneys leads to a reduction in the overall caloric load and, thus, to body weight decrement, an effect that can contribute to the management of T2DM, especially when associated with obesity or overweight. Finally, an aspect that attracted the interest of researchers toward this new antidiabetic agents was the almost exclusive expression and highly specialized function of SGLT-2 in the proximal tract of the renal tubule; consequently, highly selective inhibitors of this cotransporter were expected to not produce adverse effects on other cellular functions. Although SGLT-2 inhibitors are generally well tolerated, it was suggested that certain side effects, such as genitourinary tract infections, dehydration, potential increased risk of ketoacidosis, should be closely monitored. However, the occurrence of some other adverse reactions, such as the increased risk of bone fractures and amputation, was debated because these effects were rarely observed and are not clearly correlated to gliflozin therapeutic use.^30,31^ Overall, the incidence of adverse events ascribable to the clinical use of these drugs was low and in most cases well-controlled; therefore, it is ascertained that the risk/benefit ratio of gliflozins is favorable.^27,31^

Interestingly, gliflozins generate cardioprotective effects that can reduce the risk of cardiovascular death and hospitalization for heart failure.^6,8,27,32−36^ Preclinical assessment indicated a clear improvement of multiple cardiovascular (CV) risk factors, and, subsequently, the results of clinical trials (e.g., EMPA-REG OUTCOME, CANVAS Program, VERTIS-CV, DAPA-HF, DAPA-CKD) even exceeded expectations, showing a marked reduction in cardiac adverse events in diabetic patients with high CV risk who took gliflozins. As compared with placebo, these patients had a lower incidence of CV and renal outcomes as well as a marked reduction of hospitalization and death for heart failure; this latter effect was also observed in nondiabetic subjects, as confirmed by subsequent clinical trials.^8,9,32−40^

Several mechanisms of cardiorenal protection due to SGLT-2 inhibition were proposed.^6,11,34−36^ As reported above, the improvement of obesity/overweight, mostly the reduction of abdominal fat, leads to enhanced tissue sensitivity to insulin, counteracting the insulin resistance typical of T2DM, and results in an improvement of lipidemic profile. Body weight loss can also contribute to blood pressure reduction, although this latter is mainly determined by the natriuretic action of gliflozins and the accompanying plasma volume depletion. These actions undoubtedly have a beneficial impact on CV diseases, but it was strongly suggested that other mechanisms are involved, to explain the rapid onset of cardioprotective effects observed during therapy with gliflozins. Moreover, these cardiorenal benefits were shown to be at least in part independent of glucose-lowering effects and were observed also in the presence of reduced kidney function, a condition where the anti-hyperglycemic activity of gliflozins becomes weaker.^36^

In the course of treatment with SGLT-2 inhibitors, natriuresis and reduction of blood pressure can determine improvement in both cardiac hemodynamic and vascular function. Direct myocardial effects were also observed, such as an improvement in cardiac metabolism and bioenergetics, which were attributed to increased ketogenesis and concomitant reduction of oxidative/inflammatory stress. In addition, it was reported that SGLT-2 inhibitors can induce improvement in myocardial structure, with reduction of cardiac fibrosis and necrosis, along with changes in adipokine kinetics and epicardial adipose tissue volume.^6,35,36^ Natriuresis and reduced blood pressure can also be responsible for nephroprotective effects, mainly related to improved tubule-glomerular efficiency. It was demonstrated that therapeutic treatment with SGLT-2 inhibitors can result in improved intraglomerular hypertension and hyperfiltration, reduced albuminuria, and increased production of erythropoietin; moreover, the resulting improved renal functionality showed to slow down the progression of nephropathy. Overall, cardiorenal benefits of SGLT-2 inhibitors appeared to be the result of multifactorial interrelated mechanisms regarding both heart and kidney functions.^6,27,36^

Recent preclinical and clinical studies revealed that known SGLT-2 inhibitors were capable of reducing the expression or activation of several inflammatory mediators, such as IL-6, IL-1β, and TNF-α, resulting in anti-inflammatory effects and improving cardiovascular and immune responses. Even though further investigations are required to corroborate these findings, it was suggested that these properties could be exploited to prevent or reduce the risk of vascular and inflammatory adverse complications consequent to SARS-CoV-2 infection.^41^

Moreover, although SGLT-2 is mainly expressed in the proximal renal tubule, significant levels of this protein were also found in cerebral, breast, pancreatic, and prostate cancers, where this symporter resulted to be involved in the mechanisms of cellular glucose uptake required for tumor survival and proliferation. These recent findings suggested a new therapeutic potential for SGLT-2 inhibition and could prompt further investigations of clinically available SGLT-2 inhibitors as agents for anticancer therapy.^16,19,42^

The research on SGLT inhibitors was further boosted by the discovery that the inhibition of intestinal SGLT-1 can provide a novel opportunity to control hyperglycemia without causing serious unwanted effects and, therefore, to develop new antidiabetic candidates,^13−15,19^ as it will be discussed below. Very recent research also showed that SGLT-1 expression increased in cultured cardiomyocytes, which were treated with high glucose and lipid concentrations, inducing oxidative stress and apoptosis; this finding suggested that SGLT-1 could be implicated in glucolipotoxicity-derived cardiac damage, thus prompting further studies to investigate the possible contribution of SGLT-1 to different pathogenetic mechanisms.^43^

## 3 SGLT-1 and SGLT-2 Inhibitors: From Discovery to Development as Novel Therapeutic Agents

### 3.1 Phlorizin and *O*-Glucoside Derivatives

**Figure 1:**
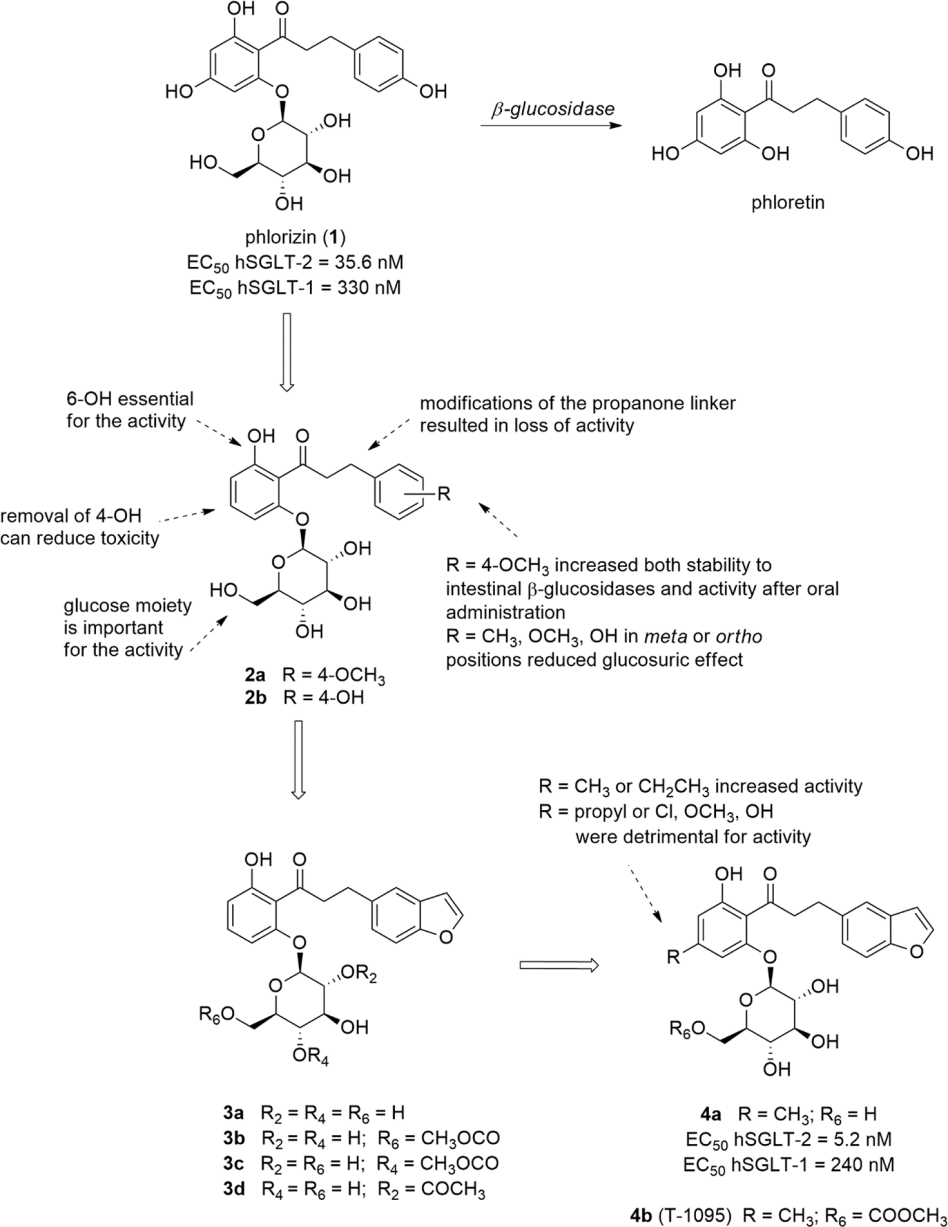
Structures of phlorizin and selected 4-dehydroxyphlorizin derivatives.

Phlorizin (**1**, Figure 1), an *O*-glucoside isolated from the root bark of the apple tree, is considered the lead compound of SGLT-2 inhibitors, although initially it was assumed as an antipyretic agent useful for the treatment of malaria.^44^ This glucoside exhibited a potent glucosuric effect in diabetic animal models, by being shown to reduce glycemic levels and normalize tissue sensitivity to insulin.^25,45^ At a later time, it was demonstrated that these effects are elicited by the inhibition of SGLT cotransporters. However, several features were considered inappropriate at the time for its further development as an antidiabetic drug, above all its moderate selectivity toward SGLT-2 versus SGLT-1 (hSGLT-2 EC_50_ = 35.6 nM; hSGLT-1 EC_50_ = 330 nM).^25,44,46^ More importantly, the metabolic lability of the *O*-glucosidic bond represented the main drawback for oral administration of phlorizin since the fast hydrolysis by intestinal β-glucosidases is responsible for both low bioavailability and toxic effects. In particular, the release of the dihydrochalcone aglycone phloretin (Figure 1) was related to strong GLUT inhibition, thus reducing cellular glucose uptake and causing damage in several tissues, including the brain.^25,44,47^

Despite these limitations, the discovery of the SGLT inhibitory activity of phlorizin was crucial for the knowledge of the physio-pathological roles of SGLTs as well as for the subsequent development of SGLT-2 inhibitors as a class of oral antidiabetic drugs.^44^ Conformational studies of phlorizin and its analogues, performed by means of NMR and molecular dynamics, led to the generation of a first pharmacophore model for SGLT inhibitors, which comprised the hydroxyl groups in positions 2, 3, 4, and 6 of the pyranoside ring, along with those in positions 4 and 6 of the proximal aromatic ring, as acceptor/donor groups for hydrogen bonding, whereas the distal phenyl ring appeared to protrude toward the edge of the binding site.^48^ More recently, docking experiments in an outward-facing model of hSGLT-1 highlighted that the glycoside ring of phlorizin is able to assume the same position of the substrate glucose, by establishing hydrogen bond interactions with several residues, such as Asn78, His83, Glu102, Tyr290, Trp291, and Lys321, while the aglycon portion is positioned into an outer vestibule protruding toward the extracellular space; in addition, the Arg267 guanidinium group can make a strong cation-π interaction with the aglycon tail. Analogous interactions were found in the hSGLT-2 model, with the difference of an additional aromatic residue His268 (included in EL5c loop), which, along with His80 and Phe98, creates an hydrophobic cage around the central benzene ring of the aglycon, by establishing additional π–π interactions and hydrophobic contacts absent in hSGLT-1.^21^

Moreover, studies on phloretin showed that the four hydroxyl groups present on both phenyl rings could be related to the toxicity of this aglycone since they appeared to be involved in the inhibition of GLUTs.^49^ In addition, the 4-OH on the proximal ring appeared to be not essential for SGLT inhibition, but it was implicated in the inhibition of Na^+^/K^+^-ATPase caused by phlorizin.^49^ Therefore, 4-dehydroxyphlorizin derivatives were designed to obtain safer analogues (Figure 1), whereas the hydroxyl group in the position 6 of the proximal benzene ring was shown to be essential for the SGLT inhibitory effect of these chalcone *O*-glucosides.^50^ In particular, Tsujihara and colleagues reported that the oral administration of compound **2a** (Figure 1) in rats (at 100 mg/kg dose) produced a marked glucosuric effect, which was 35-fold and 6-fold more potent than phlorizin and 3-(4-hydroxyphenyl) analogue **2b**, respectively.^49^ In contrast, when administered intraperitoneally (at 10 mg/kg dose), phlorizin, **2a** and **2b** produced UGE values almost similar. These findings clearly suggested that the replacement of the hydroxy group in the *para* position of the distal benzene ring with a methoxy one provided higher stability to intestinal β-glucosidases, thus markedly improving oral bioavailability of compound **2a**. In addition, both glucoside **2a** and its aglycone showed only a weak capability to inhibit GLUT-1, thus showing less toxicity than phlorizine.^49^

On the other hand, the introduction of more than one substituent group on the distal benzene ring or the displacement of a methyl, methoxy, or hydroxyl group from the *para* to the *meta* or *ortho* positions was shown to be unfavorable for the activity.^51^ Moreover, several attempts to modify the propanone linker of these chalcone-derived *O*-glucosides failed to produce beneficial effects; the replacement of the glucose moiety with different sugars also led to drastic reduction of the activity.^51^ In the course of these studies, benzofurane derivative **3a** was found to be a potent glucosuric agent, almost twice as potent as analogue **2a**, after oral administration in rats (at 100 mg/kg dose).^51^ It is worth noting that the benzofuranyl moiety can be considered a closed model of the 4-hydroxyphenyl group of phlorizin or the 4-methoxyphenyl of compound **2a**.

The introduction of a methyl or ethyl group at the 4 position of the proximal ring of compound **3a** induced the increase of glucosuric potency, whereas a propyl group or different substituents (such as Cl, OMe, OH) was related to lower or insufficient activity levels. Indeed, the oral administration of compound **4a** (Figure 1) in rats (at 100 mg/kg dose) produced a three-fold more potent glucosuric effect than analogue **3a**. When tested for its capability to inhibit SGLT activity in kidney brush border membrane vesicles prepared from tissues of both normal and diabetic rats, compound **4a** showed about two-fold higher activity than phlorizin.^52^

The corresponding 6-*O*-methoxycarbonyl-β-d-glucopyranoside prodrug **4b** (T-1095, Figure 1) was designed to increase the stability toward intestinal β-glucosidase-catalyzed hydrolysis.^50^ It emerged as a promising new lead compound due to its favorable profile in preclinical trials, being metabolized to the corresponding active SGLT inhibitor **4a** mainly by liver esterases and thus increasing both oral bioavailability and potency. Preclinical evaluation evidenced that **4b** is capable of improving glycemic control and to reduce HbA1c levels, without causing hypoglycemia, in diabetic rodents, but not in normoglycemic animals.^52^

The capacity to prevent episodes of hypoglycemia, which is an important shared characteristic of SGLT-2 inhibitors, was rationalized considering that SGLT-2 inhibition causes UGE only in the presence of hyperglycemia, when filtered glucose exceeds TmG, and the reabsorption process is saturated; under these conditions, SGLT-2 inhibitors are capable of lowering TmG and further promoting glucose excretion. On the contrary, when glycemia is normalized, TmG exceeds filtered glucose amount, even in the presence of partial SGLT-2 inhibition, and, therefore, SGLT-2 inhibitors induce neither glucosuria nor hypoglycemia.^50,52^ The ability to reduce glycemic levels without inducing hypoglycemia, which a serious side effect of certain other glucose-lowering agents, is an attractive feature of all SGLT-2 inhibitors, which has greatly contributed to their development as new antidiabetic drugs.

Long-term treatment of diabetic animals with compound **4b** induced UGE decrement, as a consequence of improved glycemic control and reduced glucotoxicity.^52^ Moreover, the oral administration of **4b** also was shown to control body weight gain and prevent the development of diabetic neuropathy in rats.^52,53^ On the basis of these preclinical results, **4b** entered clinical trials as prodrug of *O*-glycoside **4a**.

The strategy aimed to modify 2-, 4-, or 6-OH groups of the glucose moiety to achieve greater stability to intestinal β-glucosidases that provided other prodrugs. Among them, compounds **3b**, **3c** and **3d** (Figure 1) stood out because, after oral administration in rats, their glucosuric effects were higher than that of the parent compound **3a**, as a result of their increased stability to intestinal β-glucosidase hydrolysis.^54^

Despite the interesting profiles exhibited by some of these 4-dehydroxyphlorizin derivatives in several diabetes models, none of them was approved for therapeutic use, and also the clinical evaluation of the promising candidate **4b** was discontinued, due to its scarce selectivity toward SGLT-2 over SGLT-1.^55^ In fact, in this first stage of the development of SGLT inhibitors, the inhibition of SGLT-1 was considered an unfavorable feature that could be responsible for side effects, and, therefore, marked selectivity toward SGLT-2 was assumed as an essential requisite to develop safer antidiabetic drug candidates.

To overcome the limitations of phlorizin and its hydrolytic metabolite, phloretin, new compounds were designed as molecular simplification analogues, which resulted in remarkably selective effectiveness against SGLT-2. Starting from the SAR outlined for compound **4b** and related *O*-glycoside derivatives, the research focused on the effects induced by possible modifications of the structural moieties that were shown to be critical for the interaction with SGLT-2. In particular, the effects on the inhibitory potency and selectivity toward SGLT-2 compared to SGLT-1 induced by the spacer between the aglycone distal and proximal aromatic rings as well as by the hydroxyl groups of the glucoside moiety were evaluated.

**Figure 2:**
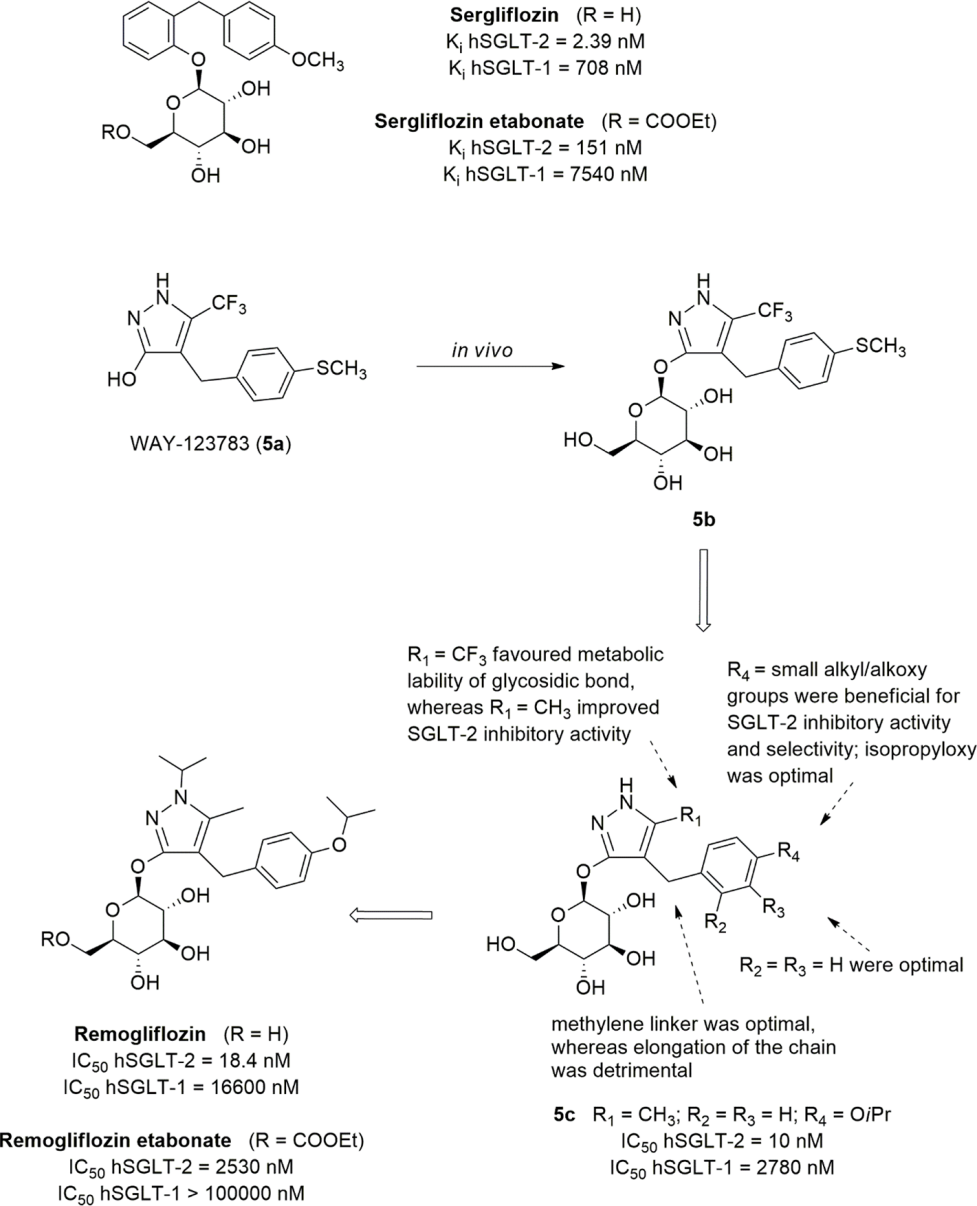
Structures and development of sergliflozin and remogliflozin.

The replacement of the propanone moiety with a shorter methylene spacer allowed the identification of a new class of benzylphenylglycoside inhibitors that proved to be effective in interacting with the SGLT-2 transporter, although Hongu et al. had found out that any change at the ketone bridge separating the two aromatic portions led to the reduction of the inhibitory effectiveness.^25,51^ This research led to the identification of sergliflozin etabonate and remogliflozin etabonate (Figure 2), which were developed as selective *O*-glycoside SGLT-2 inhibitors. To reduce the affinity for the β-glycosidase enzymes in the gastrointestinal tract, they were administered as ethyl carbonate esters (Figure 2), whose hydrolysis released the active drugs sergliflozin and remogliflozin, respectively.

Sergliflozin, the active entity of sergliflozin etabonate, is a selective inhibitor of renal SGLT-2. In healthy animals, orally administered sergliflozin increased UGE in a dose-dependent manner, determining the reduction of blood glucose in an insulin-independent way.^47^

In both healthy volunteers and T2DM patients, sergliflozin etabonate reached maximum plasma concentrations at 30–45 min after oral administration; at the tested doses, it was well tolerated without showing clinically significant adverse events.^56^ However, the duration of the glucosuric effect was limited (*t*_1/2_ 0.5–1 h), due to *O*-glycoside chemical vulnerability to hydrolysis by intestinal β-glucosidases, and therefore its development in Phase 2 clinical trials for obesity and T2DM was discontinued.^57^

To evaluate the effect exerted by the nature of the substituents of the glucoside moiety on the activity of sergliflozin as well as on the stability of its *O*-glucoside bond, a series of analogues were synthesized in which the hydroxyl groups were modified. The evaluation of UGE in rats allowed the anti-hyperglycemic capacity of the newly synthesized compounds to be assessed. The results clearly revealed that the removal or substitution of the hydroxyl groups in 2 and 3 positions of the sugar moiety were deleterious for the activity, confirming that these groups are essential for the interaction with SGLT-2. The removal of the hydroxyl groups in 4 and 6 positions brought about the reduction of activity, which is instead maintained when both groups are methylated, pointing out that the presence of the oxygen atoms in 4 and 6 is useful to establish interactions with the biological target by means of hydrogen bonds.^58^

In 2019, remogliflozin etabonate (Figure 2) was launched in India for the treatment of T2DM in adults, and, in the same year, the combination with metformin hydrochloride was commercialized.

Remogliflozin is a potent and selective SGLT-2 inhibitor, characterized by a short half-time making necessary twice-daily dosing (100 mg tablets twice daily).

The design of remogliflozin, the active entity of prodrug etabonate, was the result of merging *o*-benzylphenol *O*-glucoside sergliflozin and the 4-benzylpyrazole *O*-glucoside (**5b**), the active metabolite of prodrug **5a** (WAY-123783, Figure 2). This latter had been demonstrated to possess anti-hyperglycemic effectiveness by increasing UGE in a dose-dependent manner, when administrated to both healthy and diabetic animals, without stimulating insulin secretion nor blocking intestinal glucose absorption. However, compound **5a** was found inactive on hSGLT-2 expressed by COS-7 cells transiently transfected with hSGLT expression plasmids.^59^ Therefore, supposing that the active drug was the corresponding glucoside metabolite, *O*-glucoside **5b** was synthesized and proved to be an excellent inhibitor of SGLT-2 transporter.^25,60^

To improve the metabolic stability as well as the selectivity for SGLT-2, an extensive SAR study was performed. The discovery of remogliflozin as an SGLT-2 inhibitor required the design and synthesis of several compounds (Figure 2) which were screened for their inhibitory effects on COS-7 cells expressing both SGLT-1 and SGLT-2. Starting from previously acquired SARs, changes were designed targeted to the substituents on the vicinal aromatic ring of aglycone since the presence of electron-withdrawing groups (such as CF_3_ in compound **5b** or carbonyl group in phlorizin) appeared to favor the hydrolysis of glycosidic bond. In all cases, the elongation of methylene up to a trimethylene chain was detrimental, while a methyl group in position 5 (R_1_) proved to improve SGTL-2 inhibitory activity (such as **5c**, Figure 2).^60^ Regarding the pattern of substitution of the distal aromatic ring, an isopropyloxy group in the para position provided compound **5c** endowed with good activity and selectivity (IC_50_ hSGLT-1/IC_50_ hSGLT-2 = 278) but unfortunately lacking the appropriate oral bioavailability. Finally, the introduction of the isopropyl group on N-1 furnished remogliflozin, which showed activity similar to compound **5c** and better selectivity for SGLT-2.^60^ Interestingly, the introduction of a methyl group on N-2 reduced significantly the effectiveness, probably because its presence induces a conformation of the glucoside group inappropriate for the interaction with the target.^60^

Remogliflozin is a selective SGLT-2 inhibitor (*K*_*i*_ hSGLT-1/*K*_*i*_ hSGLT-2 = 365; IC_50_ hSGLT-1/IC_50_ hSGLT-2 = 902). Following oral administration in animal models, it showed a glucose-lowering effect by increasing UGE in a dose-dependent manner, independently of insulin levels, without increasing the risk of hypoglycemia. Moreover, it improved insulin resistance and did not induce body weight gain.^59^ In clinical trials, remogliflozin was shown to improve glycemic control in T2DM patients, even when metformin administration alone did not achieve satisfying results.^61,62^

### 3.2 C-Arylglycoside Derivatives

As discussed above, the metabolic lability of the *O*-glycosidic linkage represented a major concern in the development of *O*-glycosides as SGLT-2 inhibitors for two main reasons: (i) the hydrolysis catalyzed by intestinal β-glucosidases causes loss of activity, determining an inadequate glucosuric effect after oral administration; and (ii) unwanted side effects can occur when the released aglycone interacts with different biological targets.

**Figure 3:**
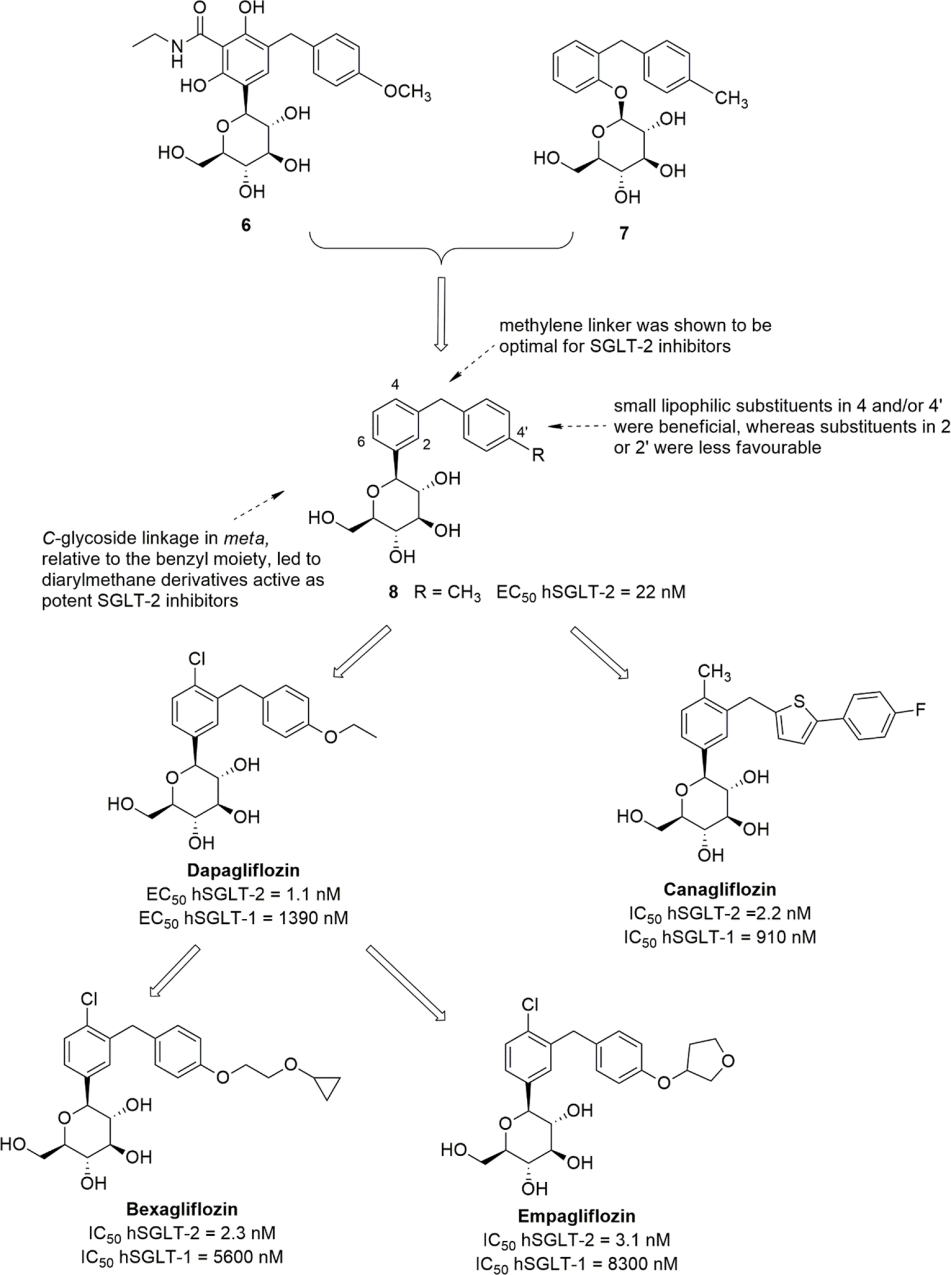
Development of *meta*-diarylmethane *C*-glucoside hSGLT-2 inhibitors.

Early efforts to generate *C*-glucosides endowed with satisfactory SGLT-2 inhibitory activity were unsuccessful; in particular, the replacement of the *O*-glycoside linkage of the dihydrochalcone derivative **3a** (Figure 1) with a methylene bridge resulted in a SGLT-2 inhibitor more than 10-fold less potent than the parent compound. Analogously, starting from *o*-benzylphenolic *O*-glucosides, the replacement of the glucoside bond oxygen with a methylene group or its removal led to a marked reduction in the affinity for the target.^25^ However, merging a *meta*-diarylmethane substituted *C*-glycosidic side product (**6**, Figure 3) with a *o*-benzylphenolic *O*-glucoside (such as **7**) provided compound **8** (Figure 3), which was the first *C*-glucoside endowed with potent SGLT-2 inhibitory activity (EC_50_ hSGLT-2 = 22 nM; selectivity versus hSGLT-1 > 600).^63^ Compound **8** exhibited 100-fold higher stability than *O*-glucoside analogue **7** in the presence of rat liver microsomes and also greater glucosuric effect in different animal models.^63^ In addition, the removal or modifications of the methylene linker between the two benzene ring of compound **8**, such as its replacement with an oxygen/sulfur atom or elongation to two/three methylene groups, caused significant reduction (from 3-fold to 29-fold) of the inhibitory effect against hSGLT-2.^63^

SAR studies revealed that, in diarylmethane derivatives, the *C*-glycoside linkage in the *meta* position of the proximal benzene relative to the distal aryl ring led to SGLT-2 inhibitors that were more effective than *ortho* or *para* isomers. Moreover, the introduction of small lipophilic substituents in positions 4 of the proximal ring and/or 4′ of the distal ring improved both activity and selectivity, whereas the substitution of the positions 2 or 2′ was detrimental (Figure 3).^25^ In addition, SGLT inhibition can be modulated by different substituents in position 6 of the proximal benzene ring; in particular, in 4-Cl-4′-alkyl/alkoxy-substituted diarylmethane *C*-glucosides, the introduction of an appropriate substituent in 6, such as an alkoxyl, haloalkyoxyl or hydroxyl group, was generally beneficial for inhibitory activity toward SGLT-2 over SGLT-1.^25,64,65^

SAR exploration carried out on diarylmethane *C*-glucosides allowed the development of dapagliflozin (Figure 3),^46^ which was the first SGLT-2 inhibitor to be approved by the European Medicines Agency (EMA), in 2012, for the treatment of T2DM. The structure of dapagliflozin includes several features that, accordingly to SAR studies, exerted a favorable influence on SGLT inhibition; in particular, two lipophilic substituents, chloro and ethoxy, in positions 4 and 4′ of the aromatic rings, respectively, were related to the potent selective inhibition of renal SGLT-2. Dapagliflozin exhibited excellent inhibitory activity toward hSGLT-2, with EC_50_ = 1.1 nM and 1263-fold selectivity over hSGLT-1 (EC_50_ hSGLT-1 = 1390 nM).^46^ In addition, very weak inhibition of GLUT-1 and GLUT-4 at 20 μM and no significant interactions with other molecular targets were detected.^25,46^ Binding studies performed with dapagliflozin and some analogues highlighted that the interactions established by means of the sugar and aglycone moieties with the respective sites of the target are mutually influenced. The binding of the aglycone was shown to be the main determinant for the affinity of SGLT-2 inhibitors; meanwhile, its orientation can be markedly influenced by the binding of the sugar, which in turn is important for the recognition of the hSGLT glucose-binding site and inhibitor selectivity.^66^ Docking of dapagliflozin into the above-mentioned outward-facing models of hSGLT-1 and hSGLT-2 suggested a binding mode similar to that described for phlorizin, with the glycoside ring positioned into the glucose-binding pocket and the aglycon tail allocated into the outer vestibule; however, compared to phlorizin, dapagliflozin appeared to be inserted deeper in the binding site, and thus its central benzene ring was able to establish critical interactions with His268 of hSGLT-2, while it is too far to strongly interact with Arg267 in hSGLT-1, thus resulting in higher SGLT-2 selectivity than phlorizin.^21^

The resulting high affinity of dapagliflozin for hSGLT-2 determined tight binding and slow dissociation of the drug from its target cotransporter.^66^ As a consequence of the metabolic stability of *C*-glucosidic linkage toward intestinal β-glucosidases, the oral administration of dapagliflozin in animal models produced a dose-dependent glucosuric effect significantly more potent than analogous *O*-glucosides, such as sergliflozin.^46^

In T2DM patients, once-daily administration of dapagliflozin for several weeks (2–24 weeks) resulted in the reduction of both fasting/postprandial glycemia and HbA1c levels, enhanced insulin sensitivity, and, on the whole, improved glycemic control, without significant risk of hypoglycemia or renal damage.^67−70^

Moreover, it was demonstrated that in these patients the glucose-lowering effect consequent to glucosuria determines a significant improvement of β-cell function, through the reduction of chronic hyperglycemia-induced glucotoxicity.^70^ Furthermore, several clinical trials demonstrated that the coadministration of dapagliflozin with other antidiabetic drugs, such as metformin or pioglitazone, in patients with T2DM not adequately treated with a single drug, can enhance glycemic control, also reducing body weight and blood pressure; once again, events of hypoglycemia were rare, and no severe episode occurred.^71,72^ Moreover, in a T2DM animal model, it was demonstrated that dapagliflozin also counteracted the progression of some chronic diabetic complications, by reducing hyperglycemia-induced inflammatory and oxidative stress in kidney and liver tissues; these findings revealed an additional interesting feature of dapagliflozin which could provide a further valuable contribution to the management of DM and its complications and promoted the start of clinical trials to assess the effects of this drug on renal and cardiovascular pathologies.^73^

Starting from the *meta*-diarylmethane *C*-glucoside **8** and the relative SAR studies, canagliflozin (Figure 3), a new *C*-glucoside analogue, was developed and approved in 2013 by EMA; in the same year, it was the first SGLT-2 inhibitor approved as an antidiabetic drug by the US-FDA. An heteroaromatic ring was inserted in the distal aryl portion, whereas the substitution pattern on the proximal benzene ring was kept nearly unchanged, with the *C*-glycosyl moiety in the *meta* position and a small lipophilic substituent in position 4, which had been shown to be related to higher inhibitory potency toward SGLT-2 over SGLT-1.^74^ Out of a series of designed heterocyclic analogues, thiophene derivatives were shown to be the most interesting, and, in particular, canagliflozin emerged as the most potent and selective hSGLT-2 inhibitor (IC_50_ hSGLT-2 = 2.2 nM; IC_50_ hSGLT-1 = 910 nM).^74^ It was capable to produce a significant increase in UGE after oral administration (at the dose of 30 mg/kg) in rats; a single 3 mg/kg oral dose significantly reduced glycemic levels in hyperglycemic mice, whereas no appreciable effect was detected in normoglycemic animals.^74^ The good pharmacological and pharmacokinetic profile shown in preclinical studies prompted the selection of canagliflozin as a candidate for clinical trials and finally its approval as a new antidiabetic drug. More recently, clinical trials (CANVAS Program) demonstrated that the administration of canagliflozin in T2DM patients can reduce the incidence of cardiovascular and renal outcomes, which represent serious DM-associated complications, although it was recommended that further long-term assessment in patients without prior cardiac events or with established kidney disease could be carried out.^38,39^ Concerns arose because of the increase of low-density lipoprotein cholesterol, which was found to be slightly higher than that observed with other SGLT-2 inhibitors.^5^ In addition, it is worth highlighting that, in CANVAS trials, canagliflozin was found to be associated with a greater risk of lower limb amputation compared with controls, whereas this adverse effect was not reported in clinical trials with other gliflozins.^31,37^

Meanwhile, considerable efforts were made to investigate new dapagliflozin derivatives. In continuation of previous SAR studies, Xu et al. demonstrated that the introduction of different substituents in position 4′ of the distal benzene ring of dapagliflozin can exert a marked influence on SGLT inhibitory effects. In particular, cycloalkoxyethoxyl groups were generally the most beneficial substituents to achieve excellent hSGLT-2 inhibition and selectivity, also providing good glucosuric effects in animal models,^75^ whereas a distal biphenyl motif slightly reduced the activity toward hSGLT-2 compared to the parent dapagliflozin.^76^ Out of the investigated dapagliflozin derivatives, bexagliflozin (EGT1442, Figure 3) emerged as a potent and highly selective hSGLT-2 inhibitor, with IC_50_ values toward hSGLT-2 and hSGLT-1 of 2.3 and 5600 nM, respectively.^75^ Therefore, the introduction of a cyclopropyloxy tail on the ethoxy group in position 4′ of the distal benzene ring did not influence SGLT-2 inhibitory potency significantly, but it appeared to be less favorable for SGLT-1 inhibition. Furthermore, the increase of the ring size up to cyclobutyl or cyclopentyl kept similar activity levels toward hSGLT-2, whereas the cycloexyl moiety resulted in lower inhibitory effectiveness.^75^ Preclinical studies revealed that bexagliflozin produced a sustained dose-dependent reduction of both plasma glucose and HbA1c levels, related to increased UGE, without inducing insulin secretion.^77^ In addition, it improved the survival of rats fed with a stroke-promoting diet.^77^ Bexagliflozin is currently under Phase III clinical investigation for the treatment of T2DM; several trials demonstrated that, in diabetic patients, also suffering from nephropathy, the oral administration of this new *C*-glucoside (at the dose of 20 mg/day) was well tolerated and allowed sustained control of both glycemic and HbA1c levels as well as reduction in body weight and blood pressure values.^78,79^

SAR studies aimed at modifying the substitution patterns on the distal benzene ring of dapagliflozin led to a new potent and selective competitive hSGLT-2 inhibitor, empagliflozin (Figure 3), which was approved for clinical use by both the US-FDA and EMA in 2014. Analogously to the insertion of a cyclopropoxyethoxyl substituent in the position 4′ of the distal benzene ring, the introduction of the tetrahydrofuran-3-oxy moiety did not modify SGLT-2 inhibitory effect significantly, but it reduced the potency against SGLT-1 (IC_50_ hSGLT-2 = 3.1 nM; IC_50_ hSGLT-1 = 8300 nM), compared to dapagliflozin (IC_50_ hSGLT-2 = 1.2 nM; IC_50_ hSGLT-1 = 1400 nM).^80^ The interesting selectivity of empagliflozin versus other transporters belonging to the SGLT family was also demonstrated and, moreover, inhibition of GLUT-1 was negligible up to 10 μM drug concentration.^80^ The administration of empagliflozin to T2DM patients (25 mg/day for 2 weeks) provided rapid and lasting effects, i.e., a significant decrease in plasma glucose concentration and glucotoxicity, along with amelioration of β-cell function.^81^ Clinical trials revealed additional beneficial actions of empagliflozin, among which was reduction of both body weight and blood pressure, and nephroprotective effects.^82,83^ The treatment of T2DM patients with different combinations of empagliflozin and metformin resulted in improved HbA1c levels and body weight reduction compared to monotherapy.^84^ Once again, as observed with dapagliflozin and canagliflozin, major hypoglycemia episodes were not detected during the treatment with empagliflozin in both monotherapy and association with insulin-sensitizing drugs.^83^

On the whole, the SAR investigation that led to the development of dapagliflozin and its *C*-glucoside analogues highlighted that a *meta*-diarylmethane-*C*-glucoside scaffold can be assumed as a privileged structural motif to obtain potent and selective hSGLT-2 inhibitors. Moreover, appropriate modifications of the distal aryl moiety can provide useful opportunities to both identify new SGLT-2 inhibitors and delineate further relevant SARs.

**Figure 4:**
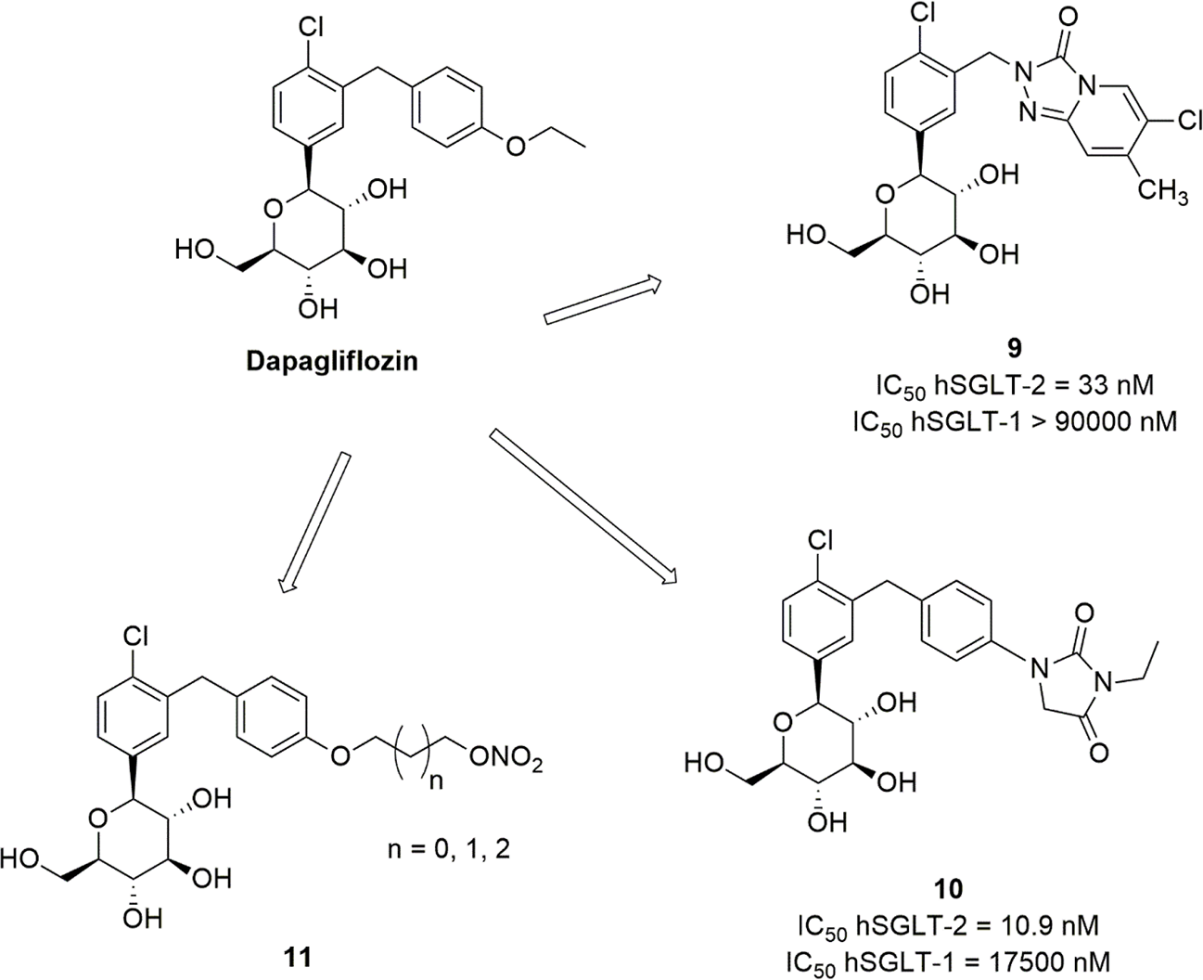
- Dapagliflozin analogues obtained by modifying the distal aryl moiety.

In this context, the distal phenyl ring of dapagliflozin was replaced by the bicyclic system [1,2,4]triazolo[4,3-*a*]pyridin-3(2*H*)-one (Figure 4). This structural modification led to a drastic reduction of SGLT-2 inhibitory potency; however, the introduction of substituents on the novel triazolopyridinone nucleus was shown to differently modulate the inhibitory effects. Derivative **9** (Figure 4), although less potent than dapagliflozin, exhibited appreciable activity and selectivity toward SGLT-2 (IC_50_ hSGLT-2 = 33 nM; IC_50_ hSGLT-1 > 90000 nM); however, it also showed low permeability, likely due to its high total polar surface area (TPSA).^85^

In addition, the small hydantoin heterocycle was introduced in position 4 of the distal benzene ring as a possible modification of the ethoxy group of dapagliflozin. Although this substitution also led to a decrement of the SGLT-2 inhibitory effectiveness compared to the parent drug (Figure 3),^46^ compound **10** (Figure 4) exhibited good activity toward the target enzyme with excellent selectivity versus SGLT-1 (IC_50_ hSGLT-2 = 10.9 nM; IC_50_ hSGLT-1 = 17500 nM). Unfortunately, although compound **10** was predicted to have appropriate ADME properties, it showed low bioavailability in animal models.^85^

More recently, to obtain new compounds with dual anti-hyperglycemic and antithrombotic activity potentially useful to prevent cardiovascular complications associated with T2DM, a modification of the ethoxy chain of dapagliflozin was performed by hybridization of dapagliflozin and a NO-donor nitrate (Figure 4). The synthesized compounds **11** showed modest SGLT-2 inhibitory effectiveness, whereas they showed antiplatelet aggregation activity attributable to an appreciable release of NO.^86^

**Figure 5:**
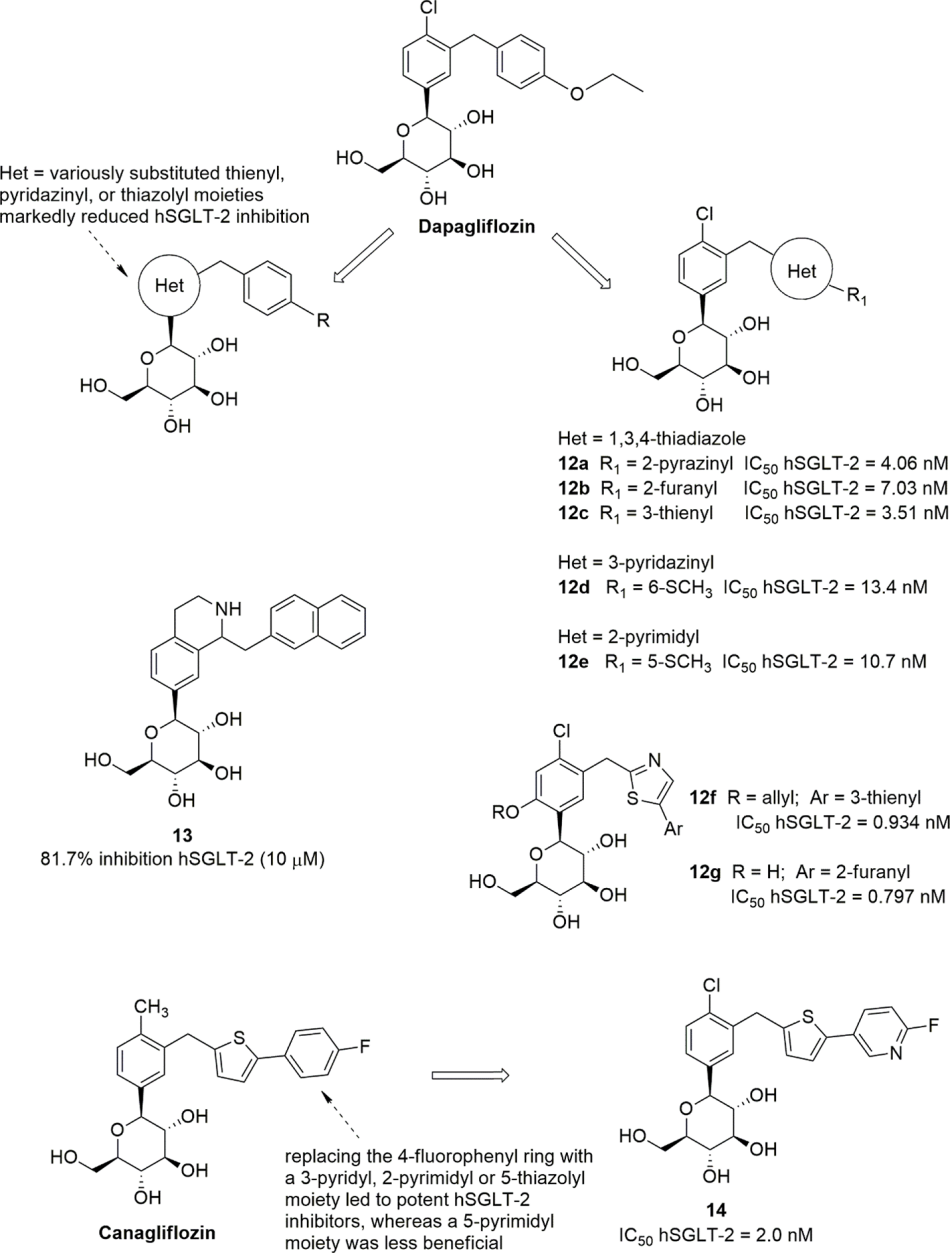
Dapagliflozin- and canagliflozin-derived aryl/heteroaryl *C*-glucosides.

Following the identification of dapagliflozin and canagliflozin as clinical candidates, several studies reported efforts aimed to obtain *C*-glucosides bearing heteroaromatic rings in either proximal or distal portions, according to the rationale that the incorporation of a heteroaryl moiety could modulate lipophilicity and selectivity (Figure 5). Lee et al. reported a series of *C*-glucosides obtained by replacing the distal benzene ring of dapagliflozin with a diaryl portion containing a 1,3,4-thiadiazole nucleus. Only compounds bearing a second heteroaromatic ring linked to position 2 of the 1,3,4-thiadiazole core, such as 2-pyrazinyl (**12a**), 2-furanyl (**12b**), or 3-thienyl (**12c**) moieties (Figure 5), exhibited in vitro inhibitory activities against hSGLT-2 in the low nanomolar range (IC_50_ < 10 nM); however, their IC_50_ values were higher than that of parent dapagliflozin (IC_50_ = 0.49 nM) in the same experimental conditions.^87^ Analogously, several pyridazinyl- and pyrimidinyl-substituted *C*-glucoside analogues, such as 6-thiomethyl-3-pyridazinyl derivative **12d** and its 5-thiomethyl-2-pyrimidinyl isostere **12e** (Figure 5), were shown to be effective hSGLT-2 inhibitors, without ever reaching the activity levels of the parent drug.^88,89^ New *C*-glucoside analogues, possessing a thiazole-containing diaryl portion, showed that the substitution at 6 position of the proximal phenyl ring generally allowed effectiveness similar to that of dapagliflozin (compounds **12f**, **12g**, Figure 5) to be reached.^90^

On the other hand, the replacement of the proximal benzene ring of dapagliflozin with variously substituted thienyl, pyridazinyl or thiazolyl moieties generally resulted in a marked decrement in hSGLT-2 inhibition, which was attributed to unfavorable electronic effects.^91,92^

More recently, a series of dapagliflozin-derived *C*-glucosides was reported in which the proximal benzene ring was replaced by a tetrahydroisoquinoline system, starting from a pharmacophore model generated by using a set of known SGLT-2 inhibitors. Most of them showed a lower ability to inhibit the target cotransporter compared to dapagliflozin, with the exception of compound **13**, which, in the tested experimental conditions, produced a hSGLT-2 inhibition percentage similar to that of the parent drug (81.7% versus 85.4%, respectively, at 10 μM concentration).^93^

On the basis of these findings, it can be argued that the central benzene ring, which according to the above-mentioned hSGLT-2 model lies embedded within the hydrophobic cage formed by His80, Phe98, and His268,^21^ is a critical structural requirement to obtain diarylmethane C-glycosides endowed with high SGLT-2 inhibitory potency. On the other hand, deeper modifications can be tolerated in the distal aryl portion, which points toward the outer region of the binding site.

In fact, other interesting hSGLT-2 inhibitors were obtained by modifying the distal aryl moiety of canagliflozin. The replacement of the distal 4-fluorophenyl ring with different heterocycles, maintaining the central thienyl core, led to effective hSGLT-2 inhibitors when the second heteroaryl ring was a 3-pyridyl, 2-pyrimidyl, or 5-thiazolyl portion, whereas a 5-pyrimidyl moiety appeared to be less beneficial. Out of these, 3-[5-(6-fluoro-3-pyridyl)-2-thienylmethyl]phenyl substituted analogue **14** (Figure 5) emerged as an isostere as potent as the lead compound and was selected for further studies; it produced considerably increased UGE and a glucose-lowering effect in mice, along with appropriate pharmacokinetics.^94^ Studies with compound **14** (TA-3404) were also performed to assess whether SGLT-2 inhibitors can act on their renal target extracellularly, after glomerular filtration, as previously shown for phlorizin, or if they act intracellularly after entering the tubular cells. Compound **14** proved to function as an extracellular inhibitor of SGLT-2-mediated glucose transport, first being filtered in the renal glomerulus and then acting at the luminal membrane of tubule, whereas it was ineffective from the intracellular compartment.^95^

**Figure 6:**
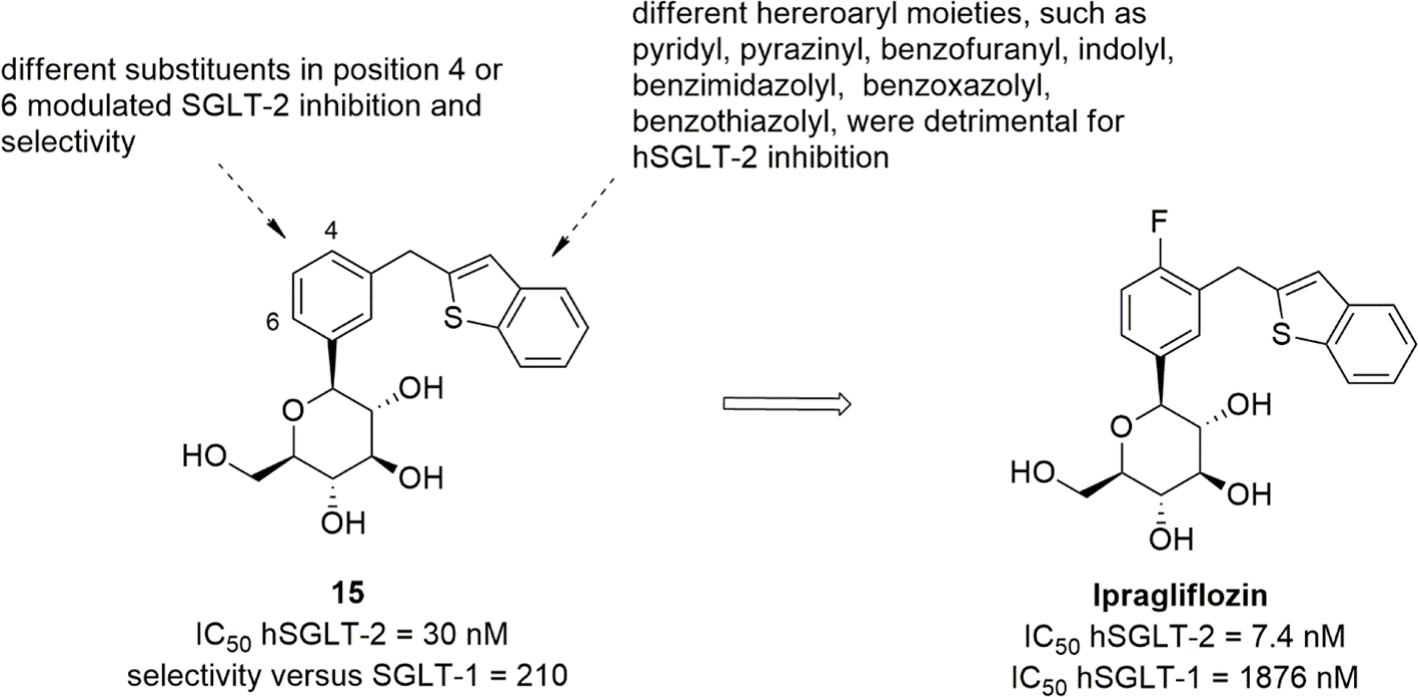
Development of ipragliflozin.

Ipragliflozin, a *C*-glucoside approved as drug in 2014 only in Japan and in a limited number of countries outside Europe and North America, emerged from the optimization of a series of *C*-glucosides containing various heteroaryl moieties.^96^ Starting from the promising benzothiophene derivative **15** (Figure 6), the introduction of a fluorine substituent in position 4 of the central benzene ring improved both hSGLT-2 inhibitory activity and selectivity over hSGLT-1, leading to ipragliflozin which exhibited an IC_50_ value of 7.4 nM toward hSGLT-2 and more than 250-fold selectivity versus hSGLT-1.^96^ The displacement of the fluorine atom in position 6, as well as the introduction of a methoxy or hydroxyl group in 4 or 6 of the proximal benzene ring, reduced the SGLT-2 inhibitory effect; on the other hand, the replacement of the fluorine substituent of ipragliflozin with a chlorine atom provided a two-fold more potent hSGLT-2 inhibitor, which, however, showed lower selectivity over hSGLT-1.^96^

Ipragliflozin showed pharmacological features shared with the previously approved SGLT-2 inhibitors, such as the stability to intestinal β-glucosidases and the capability to induce prolonged dose-dependent increase of UGE, after single oral dose administration in diabetic animals (at doses ranging from 0.1 mg/kg to 1 mg/kg).^97^ The reduction of glycemic levels was not associated with hypoglycemia risk or increased insulin secretion.^96,97^ Clinical evidence demonstrated that ipragliflozin induces sustained control of glycemic and HbA1c levels, also reducing body weight.^98^ However, extended assessment of cardiorenal effects of ipragliflozin has not been accomplished yet; in addition, it was suggested that further studies on the long-term safety profile should be performed.^98^ As the other approved SGLT-2 inhibitors, ipragliflozin is indicated for the management of T2DM in monotherapy or in combination with other antidiabetic drugs; moreover, in 2018 it was approved in Japan for the treatment of T1DM in combination with insulin.^98^

**Figure 7:**
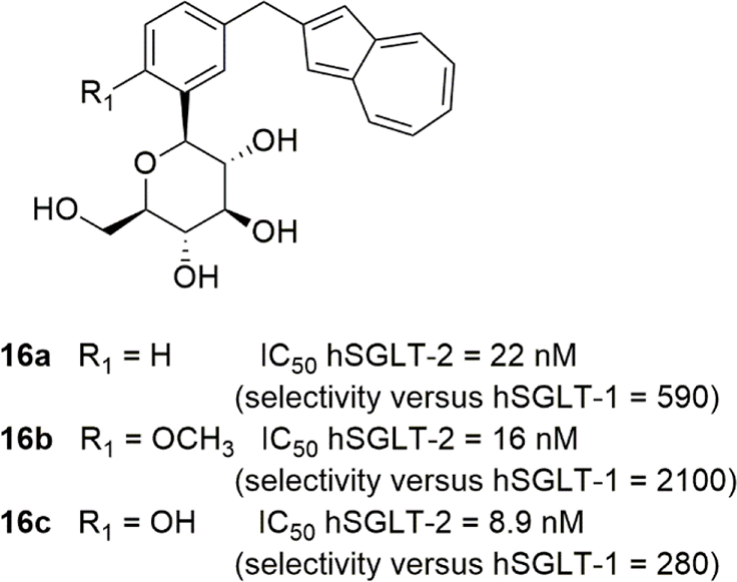
Structures of selected 3-[(azulen-2-yl)methyl]phenyl *C*-glucosides.

Novel 3-arylmethylphenyl-*C*-glycoside derivatives were synthesized by Ikegai and colleagues by replacing the distal phenyl or heteroaryl moiety with the bioisostere azulene motif. This particular substitution led to the identification of azulen-2-yl derivative **16a** (Figure 7), endowed with appreciable SGLT-2 inhibitory effectiveness (IC_50_ = 22 nM) and 590-fold selectivity over SGLT-1.^99^ Furthermore, the introduction of appropriate substituents in position 6 of the proximal benzene ring enhanced SGLT-2 inhibition, especially in 6-methoxy substituted derivative **16b** (Figure 7, IC_50_ hSGLT-2 = 16 nM, with 2100-fold selectivity over SGLT-1) and 6-hydroxyl analogue **16c** (Figure 7, IC_50_ hSGLT-2 = 8.9 nM, with 280-fold selectivity over SGLT-1). This latter was selected for further preclinical investigation, which revealed a potent and long-lasting anti-hyperglycemic activity in diabetic animal models, without implicating a hypoglycemic effect. On this basis, the choline salt of **16c** (YM543) was selected as a candidate for clinical evaluation.^99^

**Figure 8:**
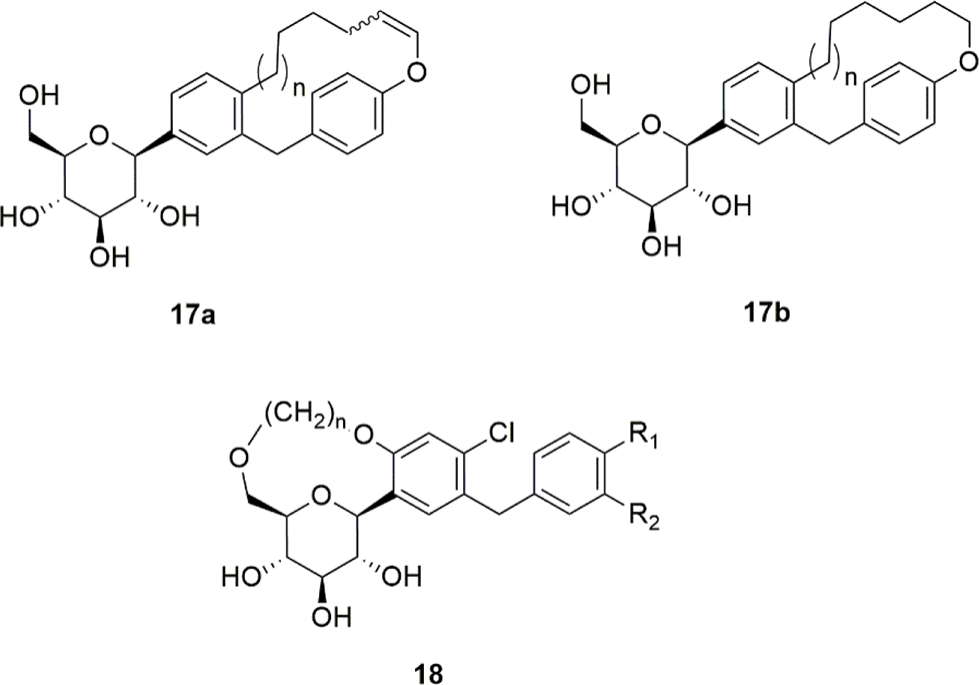
General structures of macrocyclic *C*-glycoside derivatives.

Taking into consideration the moderate SGLT inhibitory effects of acerogenins, cyclic diarylheptanoids isolated from the bark of *Acer nikoense*, an “ansa” motif connecting positions 4 and 4′ of dapagliflozin was inserted, leading to macrocyclic ether derivatives **17a,b** (Figure 8); however, the IC_50_ values of these novel analogues were at least 44-fold higher than that of the lead compound.^100^ Other macrocyclic *C*-glycosides were obtained by connecting position 6 of the proximal ring of dapagliflozin with the 6-OH of glucose (**18**, Figure 8); out of these, several compounds provided potent in vitro hSGLT-2 inhibition, but their poor pharmacokinetic properties determined modest in vivo activity.^101^

**Figure 9:**
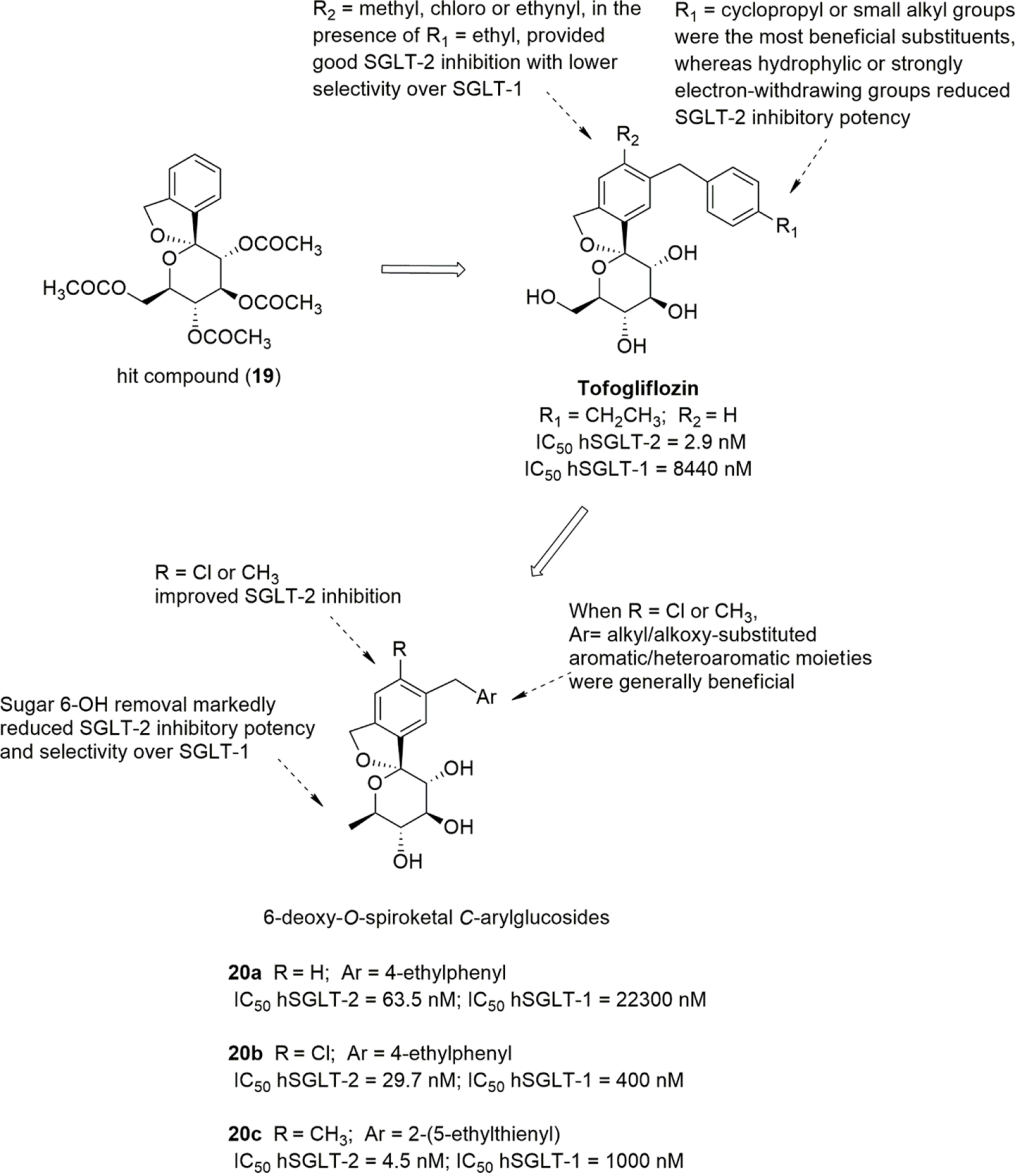
Design and SARs of tofogliflozin and derivatives.

A series of novel *O*-spiroketal-*C*-arylglucosides was designed on the basis of a 3D pharmacophoric model generated by the superposition of known inhibitors, both *O*-glucosides (such as phlorizin, sergliflozin, and remogliflozin) and *C*-glucosides (such as dapagliflozin and canagliflozin).^102^ This pharmacophore comprised two aromatic moieties and a sugar ring, whose positioning at appropriate distances were critical for the achievement of interesting SGLT-2 inhibitory activity. Database searching by using two pharmacophore features, i.e., the central aromatic moiety and the linked sugar ring, resulted in the identification of hit compounds characterized by a spiroketal scaffold (such as compound **19**, Figure 9).

Therefore, on this basis, a series of new *O*-spiroketal *C*-arylglucosides was synthesized, by introducing a *p*-substituted benzyl group in position 3 of the proximal benzene ring, analogously to previously approved glucosides.^102^ Cyclopropyl or small alkyl groups were shown to be the most beneficial substituents in position 4′ of the distal phenyl ring, whereas hydrophilic or strongly electron-withdrawing substituents were detrimental for hSGLT-2 inhibition. The introduction of a small lipophilic substituent, such as a chloro, methyl, or ethynyl group, in position 4 of the proximal benzene ring improved the hSGLT-2 inhibitory effect, although enhanced hSGLT-1 inhibition and thus reduced selectivity.^102^ Out of these novel *O*-spiroketal derivatives, tofogliflozin (Figure 9) stood out as one of the most potent and selective hSGLT-2 inhibitors (IC_50_ hSGLT-2 = 2.9 nM; IC_50_ hSGLT-1 = 8444 nM); its oral administration in diabetic mice provided a dose-dependent reduction of glucose blood levels by increasing UGE, whereas hypoglycemia was not detected. On the basis of these preclinical results and its favorable pharmacokinetic profile, tofogliflozin was selected as a clinical candidate and was approved in Japan. In clinical trials, tofogliflozin produced significant dose-dependent reduction of both fasting/postprandial glycemic and HbA1c levels as well as body weight loss, without causing severe adverse effects.^103,104^

Starting from tofogliflozin, a subsequent investigation led to 6-deoxy-*O*-spiroketal-*C*-arylglucosides, designed to assess the critical role played by the 6-OH group of the sugar moiety in hSGLT-2 recognition and the influence exerted by physicochemical properties on SGLT-2 inhibition and pharmacokinetic.^105^ Glucose 6-OH removal caused a generally remarkable reduction of the hSGLT-2 inhibitory effectiveness; indeed, the 6-deoxyglucose analogue of tofogliflozin (compound **20a**, Figure 9) showed an IC_50_ hSGLT-2 value 22-fold higher than that of the parent drug. The introduction of a substituent in position 4 of the central benzene ring, preferably a chloro or methyl group, improved the hSGLT-2 inhibitory activity. However, it is worth highlighting that the presence of 4-Cl substituent remarkably reduced SGLT-2/SGLT-1 selectivity; in fact, analogue **20b** (Figure 9) showed a 2-fold higher SGLT-2 inhibitory activity and also a markedly increased (56-fold) potency against SGLT-1 compared to the parent compound **20a**. In addition, in the presence of 4-Cl or 4-CH_3_, the most beneficial distal aryl moieties were generally shown to be alkyl/alkoxy substituted heteroaromatic rings. These features, present in compound **20c**, were shown to improve hSGLT-2 inhibitory potency (IC_50_ = 4.5 nM) and provided 216-fold selectivity over hSGLT-1. Interestingly, in animal models this compound produced higher UGE values (after a single oral administration of a 1 mg/kg dose) and better oral glucose tolerance than tofogliflozin, most likely due to a more favorable pharmacokinetic profile.^105^

### 3.3 Sugar-Modified C-Arylglycosides

**Figure 10:**
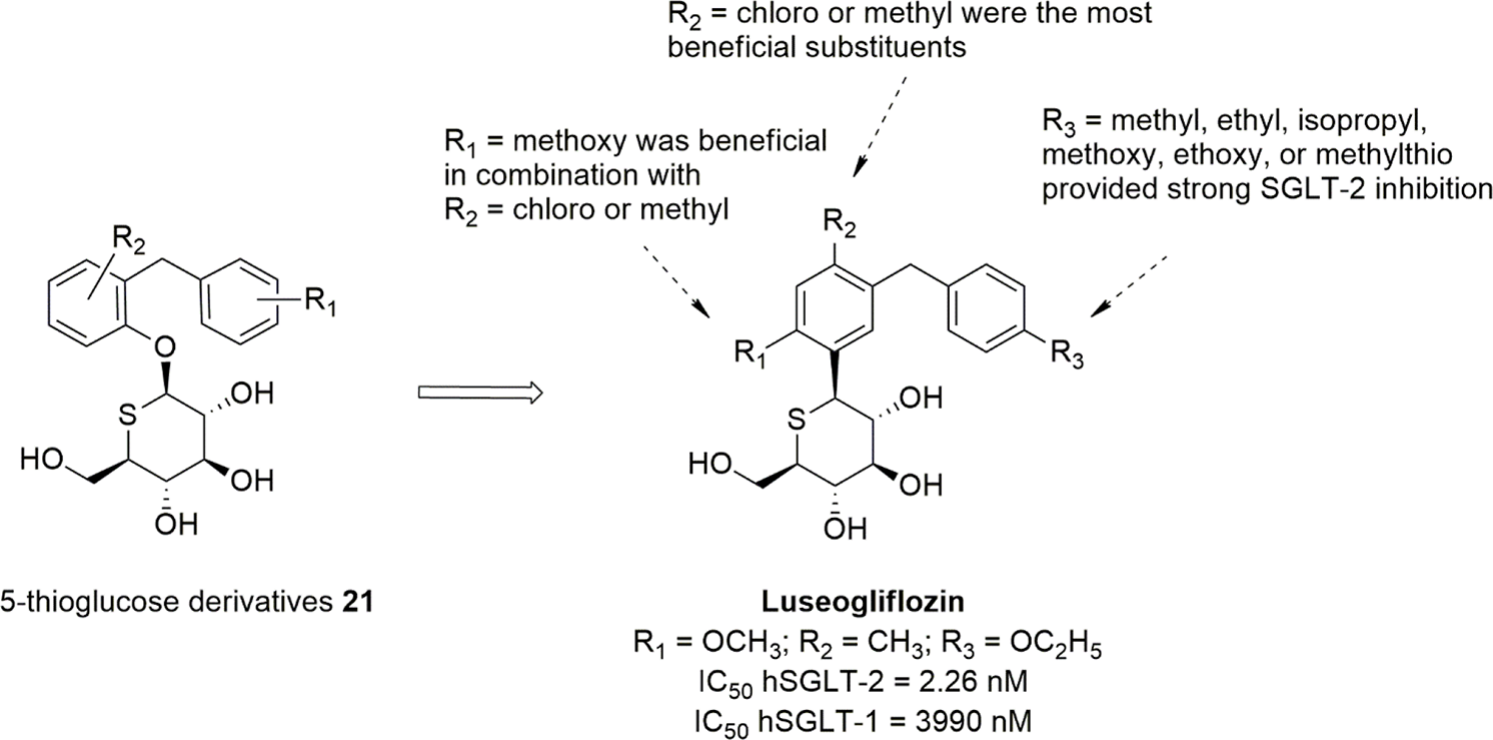
Design of luseogliflozin.

Luseogliflozin (Figure 10), a novel 1-thio-d-glucitol, was approved in 2014 in Japan for the treatment of T2DM. The initial design was aimed to obtain metabolically stable *O*-glycosides active as SGLT-2 inhibitors (compounds **21**, Figure 10), and the development of this research led to the synthesis of more effective and stable *C*-glucoside analogues (Figure 10).^106^ The optimization of the SGLT-2 inhibitory effectiveness and selectivity of these compounds was related to the combination of different substituents in positions 4 and 6 of the proximal benzene ring and in position 4′ of the distal phenyl moiety. In particular, compounds bearing a methylthio group or small alkyl/alkoxy substituents (methyl, ethyl, isopropyl, methoxy, ethoxy) in position 4′ of the distal benzene ring (R_3_) generally induced interesting SGLT-2 inhibition, and a chloro or methyl substituent in position 4 of the proximal benzene moiety (R_2_) exerted a beneficial influence. When R_2_ was a chloro or methyl group, the introduction of a methoxy substituent in position 6 of the same benzene ring (R_1_) also led to potent SGLT-2 inhibitors with improved selectivity.^106^ Luseogliflozin was shown to be a strong and selective hSGLT-2 inhibitor (IC_50_ = 2.26 nM; 1765-fold selectivity over hSGLT-1), by acting through a competitive mechanism.^106^ These results indicated that the thioglucose moiety can effectively act as a bioisostere of glucose in SGLT-2 inhibitors.

Interestingly, Uchida and colleagues performed kinetic and binding studies demonstrating that the complex luseogliflozin/hSGLT-2 is relatively stable, with a dissociation half-time of approximately 7 h, versus 60 min of empagliflozin, a slow-dissociating SGLT-2 inhibitor, and 24 s of phlorizin. This behavior, along with the higher concentration of the drug detected in kidney compared to the plasma at 4 h after oral administration in animal models, can provide a rationalization for the prolonged duration of luseogliflozin effectiveness in increasing UGE and controlling hyperglycemia.^107^ Accordingly, clinical evaluation revealed that the dose-dependent glucosuric effect of luseogliflozin was maintained for at least 48 h after a single dose administration, at all tested doses, even when its plasma concentration was low.^108^

**Figure 11:**
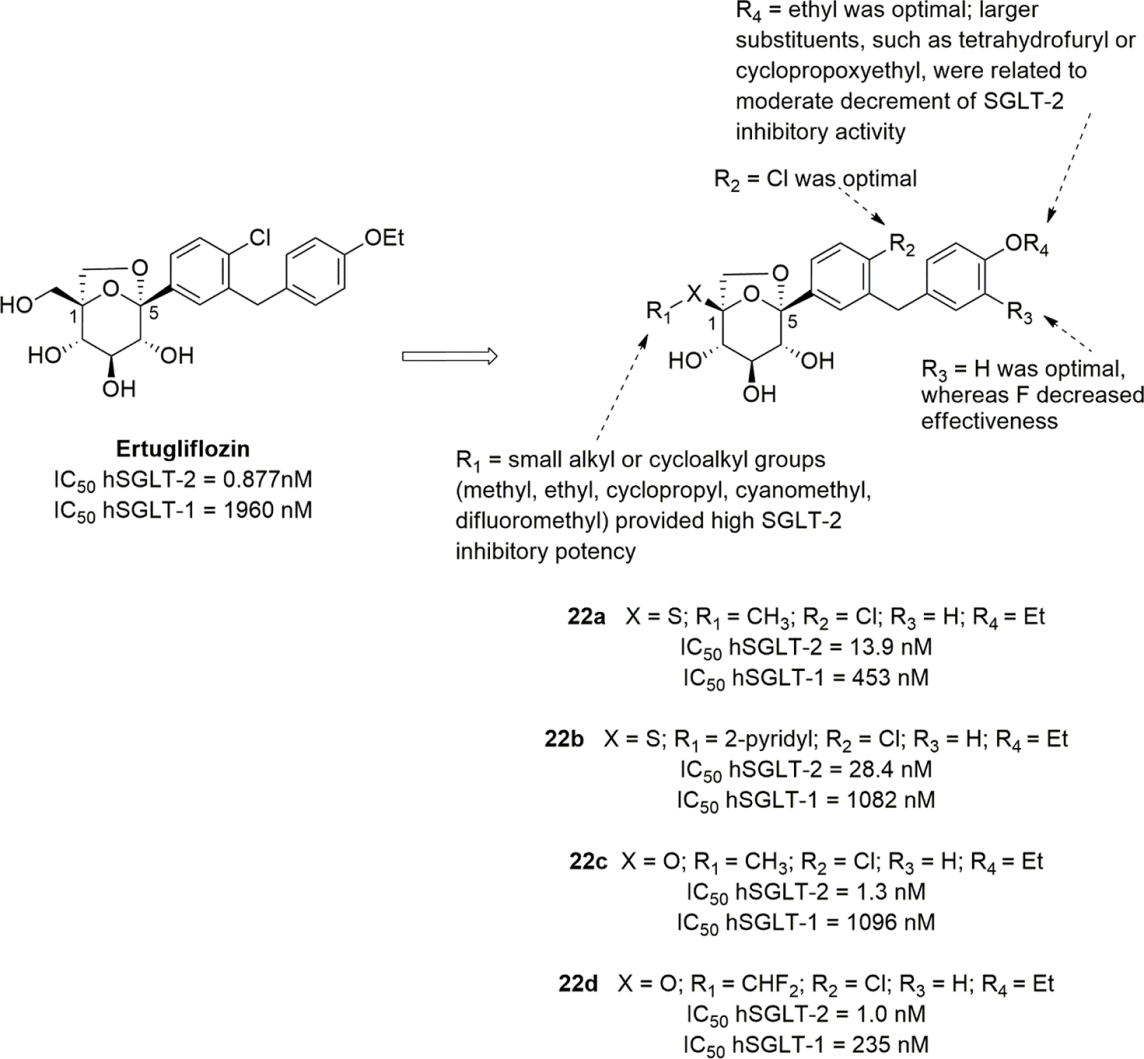
SARs of ertugliflozin-derived SGLT-2 inhibitors.

Ertugliflozin (Figure 11) is another novel SGLT-2 inhibitor derived by structural modifications of the sugar moiety; its excellent pharmacological profile led to its approval for the treatment of T2DM in USA and Europe in 2017 and 2018, respectively. It belongs to a novel series of SGLT-2 inhibitors designed by Mascitti and colleagues in the course of a research aimed to obtain compounds endowed with longer half-life and, thus, to achieve optimal daily UGE at doses as low as possible.^109^ The Authors hypothesized that the presence of an H-bond donor group at the C-5 of the sugar ring represented a critical structural feature in order to achieve this goal. In this view, the dioxa-bicyclo[3,2,1]octane motif was selected as a rigid analogue of glucose which might be favorable to enhance SGLT-2 inhibitory potency.^109^

Out of the investigated series, ertugliflozin (Figure 11) emerged as a potent and selective hSGLT-2 inhibitor (IC_50_ hSGLT-2 = 0.877 nM; IC_50_ hSGLT-1 = 1960 nM), which exhibited an excellent pharmacokinetic and safety profile and was able to provide a significant and sustained glucosuric effect in rats.^109^

Several international multicenter clinical trials (VERTIS program) demonstrated the effectiveness of ertugliflozin, as both monotherapy and combination with other oral antidiabetic agents (such as glimepiride, sitagliptin, metformin), in reducing glycemic and HbA1c levels, in a dose-dependent manner, and also controlling body weight and blood pressure in T2DM patients; in line with the other approved SGLT-2 inhibitors, low incidence of adverse effects or hypoglycaemia episodes were reported.^110−113^ An ertugliflozin-induced UGE increase was maintained after multiple doses. After a single oral administration (at the dose of 25 mg), a rapid absorption of ertugliflozin was observed, along with a high percentage (94% in humans) of drug bound to plasma proteins. In addition, the elimination half-life was about 17 h, justifying the prolonged action of ertugliflozin and its once-daily dosing.^110^ In addition, a significant improvement of glycemic control in T2DM patients inadequately controlled by metformin was observed.^112,114^

Recently, Li et al. reported the results of a SAR investigation of ertugliflozin analogues performed by exploring the effects exerted on the activity by the substitution pattern on both diarylmethane portion and sugar C-5. These new derivatives were designed as hybrids obtained by merging the dioxa-bicyclo[3,2,1]octane glycoside portion of ertugliflozin with that of sotagliflozin (see below), which is characterized by a 6-methylsulfanyloxane-3,4,5-triol moiety. However, the introduction of a sulfur atom on C-1 of the dioxa-bicyclo[3,2,1]octane motif (X = S, compounds **22a** and **22b**, Figure 11) appeared to be detrimental, leading to derivatives with SGLT-2 affinity and selectivity 15–30-fold lower than that of ertugliflozin, whereas its replacement with an isostere oxygen atom significantly enhanced SGLT-2 inhibitory potency.^115^ The presence of small alkyl or cycloalkyl groups (R_1_) on the oxygen atom in 1 (X=O) of the bicyclic moiety, such as methyl, ethyl, cyclopropyl, cyanomethyl, difluoromethyl (e.g., compounds **22c** and **22d**, Figure 11), was well-tolerated and related to excellent SGLT-2 inhibition levels (IC_50_ values ranging from 1.0 nM to 4.4 nM), whereas a hydroxyethyl group provided from 24-fold to 30-fold lower effectiveness compared to compounds **22c** and **22d**, respectively. With regard to the substitution pattern on the diarylmethane portion, R_2_ = Cl and R_4_ = ethyl (Figure 11) were shown to be optimal. When R_4_ was a substituent larger than the ethyl group, the SGLT-2 inhibitory activity moderately decreased. Out of this series, compounds **22c** (IC_50_ hSGLT-2 = 1.3 nM; IC_50_ hSGLT-1 = 1096 nM) and **22d** (IC_50_ hSGLT-2 = 1.0 nM; IC_50_ hSGLT-1 = 235 nM) (Figure 11) stood out for their high SGLT-2 inhibitory potency and capability to provide a long-lasting glucosuric effect in animal models, similar to that of dapagliflozin, and were selected for further preclinical evaluation.^115^

**Figure 12:**
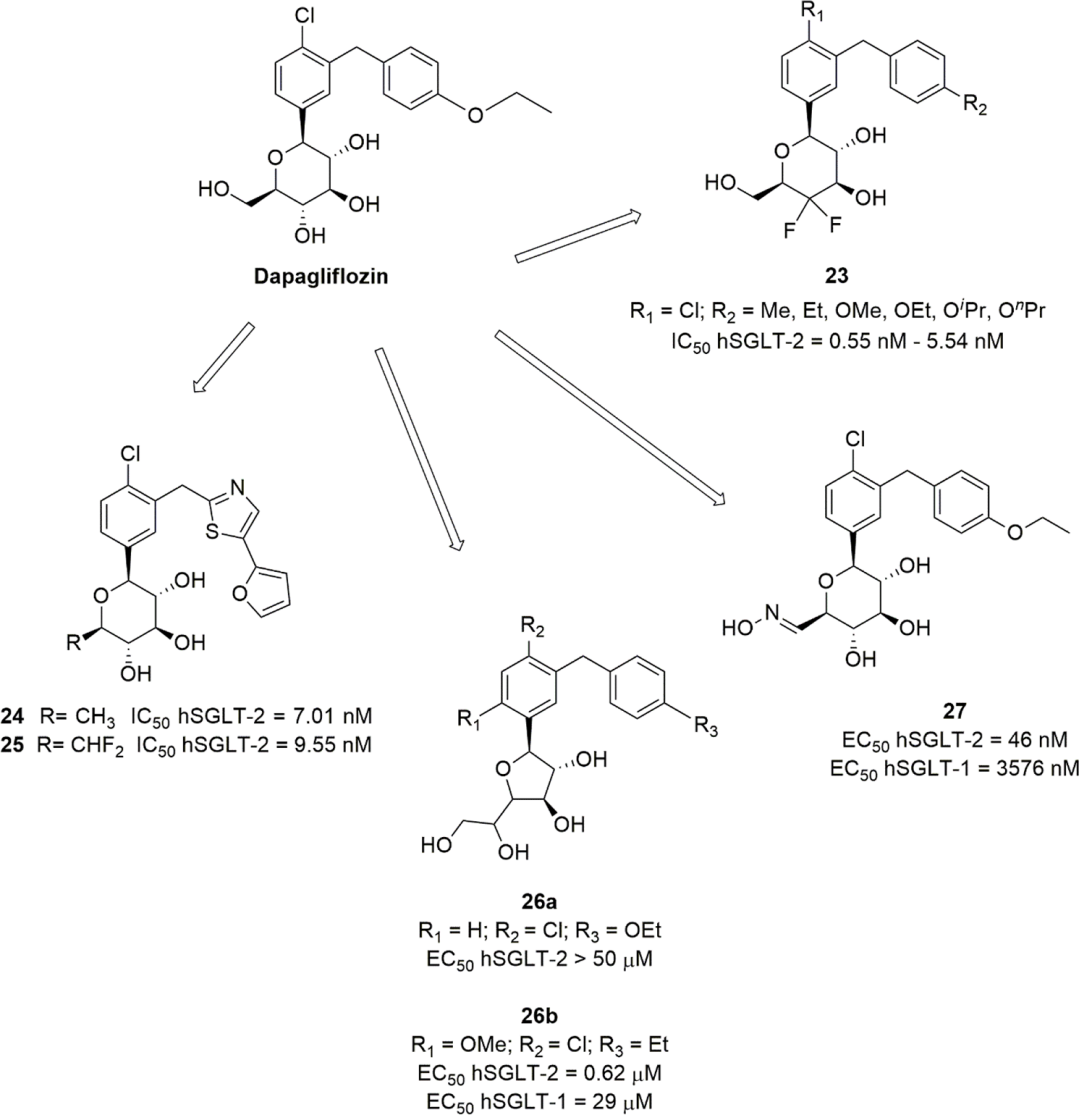
Examples of sugar-modified dapagliflozin derivatives.

Several other attempts to modify the sugar moiety of established SGLT-2 inhibitors, especially dapagliflozin, were reported. An example was the synthesis of dapagliflozin derivatives in which a *gem*-difluoro substitution was introduced in position 4 of the sugar ring (compound **23**, Figure 12). Good SGLT-2 inhibitory potency (IC_50_ ranging from 0.55 nM to 5.54 nM) comparable to that of the parent drug was achieved when the substituent in 4 of the proximal benzene ring was Cl and in position 4′ of the distal ring was introduced a small alkyl or alkyloxy group.^116^

Several structural modifications on the C-6 of the sugar moiety were carried out in thiazole-containing analogues of dapagliflozin, revealing that modifications in this position generally caused reduction of the SGLT-2 inhibitory effectiveness, especially when the hydroxymethyl group was replaced by sterically hindered substituents, such as branched or unsaturated hydroxyalkyl or thioalkyl groups. However, the removal of 6-OH or the replacement of CH_2_OH with a difluoromethyl group (compounds **24** and **25**, respectively, Figure 12) maintained appreciable SGLT-2 inhibitory activity, even if these modifications did not ameliorate the potency compared to dapagliflozin.^117^

Furthermore, dapagliflozin-derived d-glucofuranosides were synthesized to assess the effect exerted on SGLT-2 inhibition by the furanosic form of the glucose moiety.^118^ While the furanoside analogue of dapagliflozin was shown to be inactive (**26a**, IC_50_ > 50 μM) compared to the parent drug, the replacement of the ethoxy group on the distal phenyl ring with an ethyl and the simultaneous introduction of a methoxy in the position 6 of the proximal phenyl ring led to the most effective SGLT-2 inhibitor of this series (compound **26b**, Figure 12) with an IC_50_ value of 0.62 μM toward hSGLT-2 and 47-fold selectivity over hSGLT-1.^118^ However, all tested glucofuranosides turned out to be markedly less potent SGLT-2 inhibitors than glucopyranosides, revealing that the pyranose ring is required to effectively inhibit SGLT symporters through an optimal adaptation to their glucose binding site.

Dapagliflozin was also used as a template for the design and synthesis of analogues obtained by incorporating an oxime or hydrazone tail at the glycosyl C-6.^119^ The presence of the C=N linkage at this position, as well as the C–N linkage in the corresponding reduction products, produced less potent hSGLT2 inhibitors, compared to the parent drug; however, several of them showed good in vitro inhibition and selectivity levels. Out of them, compound **27** (Figure 12, IC_50_ hSGLT2 = 46 nM, with 78-fold selectivity over hSGLT-1) was selected for its promising pharmacokinetic behavior in animal models; after oral administration in rats, it induced a glucosuric effect and reduction in glycemic levels comparable with dapagliflozin.^119^

On the whole, these findings evidenced that only few modifications of the glycoside moiety are tolerated to maintain high SGLT-2 inhibitory potency and selectivity, once again highlighting that this structural portion is crucial for the interaction with the target protein.

**Figure 13:**
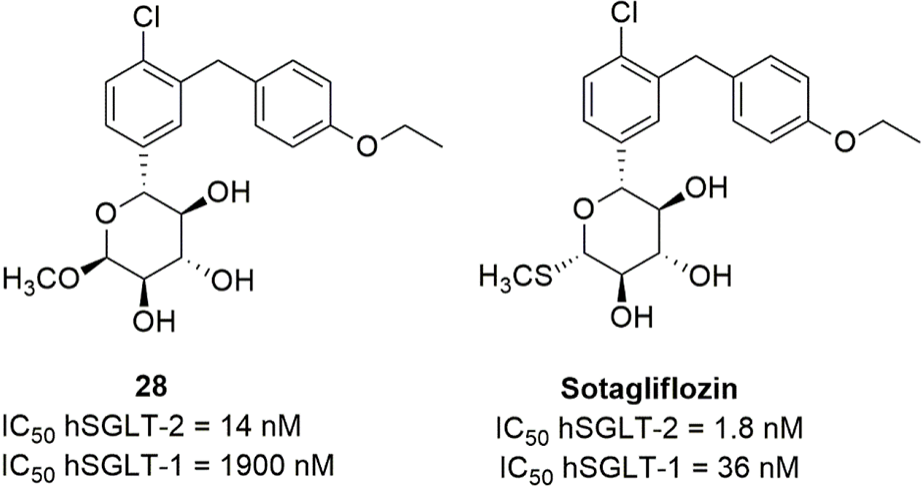
Structures of representative xylose-derived SGLT inhibitors.

In search for potent SGLT-2 inhibitors, the replacement of the d-glucopyranose moiety with l-xylopyranose appeared as an attractive variation of the sugar scaffold of aryl-*C*-glycosides. Goodwin and colleagues chose this unnatural sugar moiety to obtain SGLT-2 inhibitors endowed with higher metabolic stability and to prevent undesired cross-reactivity with other glucose-binding enzymes.^120^ Several of the synthesized novel l-xyloside derivatives were shown to be effective in vitro hSGLT-2 inhibitors. Among them, compound **28** (Figure 13) was the most active hSGLT-2 inhibitor, 343-fold more potent than its 6-epimer. It was found that variations of the methylene linker or the substituents on the benzene rings, as well as changes in the stereochemistry or substitution pattern of the sugar scaffold, led to generally marked decrement of SGLT-2 inhibitory effectiveness.^120^ In vivo, compound **28** showed significant activity, providing a dose-dependent glucosuric effect after oral administration of both single and repeated daily dosing in diet-induced obese mice; interestingly, a single oral dose of **28** (in the range 10–100 mg/kg) resulted in sustained glucosuria beyond 24 h in this animal model, suggesting a therapeutic potential for chronic management of hyperglycemia.^120^

### 3.4 Dual SGLT-1/SGLT-2 Inhibitors

In the first stage of the development of SGLT inhibitors as antidiabetic drugs, the selectivity toward renal SGLT-2 was considered an important feature required to develop safe drug candidates, taking into consideration the GGM syndrome present in SGLT-1-deficient humans. More recently, it emerged that the simultaneous inhibition of both SGLT-1 and SGLT-2 might contribute to reduce the tubular reabsorption of glucose and, thus, be beneficial to improve the glycemic control in T2DM. Studies carried out on SGLT-1 or SGLT-2 knockout (KO) mice and SGLT-1/SGLT2-double-KO (DKO) mice showed that DKO mice had higher UGE values and improved glycemic control, compared to SGLT-2 KO mice, and turned out to be healthy when maintained on glucose-free, high-fat diet.^121^ Even though SGLT-2 is the major transporter responsible for glucose reabsorption in the renal tubule, SGLT-2 KO mice showed UGE values that were 30% of the maximum UGE measured in DKO mice, suggesting that, in the kidneys, in the absence of SGLT-2, the SGLT-1 subtype can play a compensatory role by reabsorbing up to 70% of filtered glucose that is normally reabsorbed by SGLT-2 isoform. These findings supported the hypothesis that inhibiting both renal SGLT-1 and SGLT-2 could provide improved therapeutic treatment of T2DM, especially in patients with poor glycemic control and, therefore, prompted the development of dual SGLT-1/SGLT-2 inhibitors.^121−124^

The first dual SGLT-1/SGLT-2 inhibitor approved for clinical use was sotagliflozin (LX4211, Figure 13), which was assumed as an innovative lead compound to develop multitarget antidiabetic drugs. Sotagliflozin was obtained by replacing the 6-methoxy substituent of compound **28** with the isostere thiomethyl group. It proved to be more potent toward both SGLT subtypes and less selective, showing 20-fold SGLT-2/SGLT-1 selectivity (IC_50_ hSGLT-2 = 1.8 nM; IC_50_ hSGLT-1 = 36 nM), compared to parent **28** (134-fold selective toward SGLT-2 over SGLT-1).^124^

Despite the considerable capacity to inhibit both hSGLT-1 and hSGLT-2 at low nanomolar concentrations, it was shown that the clinically significant glucosuric activity of sotagliflozin is a consequence of the inhibition of renal SGLT-2, whereas the inhibition of renal SGLT-1 did not appear to elicit any appreciable effect. In fact, in T2DM patients, sotagliflozin-induced glucosuria was comparable to that of more selective SGLT-2 inhibitors; moreover, once it reached a plateau (UGE about 60 g/24 h with 200 mg daily dose), UGE values no longer increased by successive dose increments. Interestingly, clinical trials revealed that increasing doses of sotagliflozin resulted in a dose-dependent improvement of glycemic control, by reducing HbA1c, fasting and postprandial glycemic levels, without increases in UGE values, demonstrating that the mechanism of action of this drug is more complex than expected, and the contribution of intestinal SGLT-1 inhibition is crucial for its anti-hyperglycemic efficacy.^122,124−126^

Indeed, it was demonstrated that the mechanism of action of sotagliflozin involves not only the inhibition of SGLT-1 at the extracellular intestinal luminal side but also more complex downstream events.^122,125^ In both animals and humans treated with orally administered sotagliflozin plus a glucose load, a lasting increase in the circulating levels of glucagon-like peptide 1 (GLP-1) and peptide YY (PYY) was observed,^122,124,127,129,130^ showing that the reduced glucose absorption consequent to intestinal SGLT-1 inhibition can induce incretin release from enteroendocrine cells and therefore corroborating that this is a central event in the action of sotagliflozin. Indeed, the multiple actions of incretins, ranging from the control of appetite to increased insulin release and tissue sensitivity, can effectively contribute to the improvement of glycemic control. Accordingly, this effect was also observed in SGLT-1 KO mice but not in SGLT-2 KO mice.^121,122,131^ In addition, the release of GLP-1 could also be promoted by short-chain fatty acids which derive from unabsorbed glucose fermentation by microbiota in the colon.^121,125^

Interestingly, the reduction of postprandial glycemic levels induced by sotagliflozin was also shown in T2DM patients with renal impairment (estimated glomerular filtration rate eGFR < 45 mL/min/1.73 m^2^), who usually show a decrement of UGE values; in addition, a clinically relevant reduction in blood pressure was also observed in this clinical trial.^127^ On the whole, these results suggested that dual SGLT-1/SGLT-2 inhibitors, such as sotagliflozin, can provide a new therapeutic tool for the treatment of T2DM patients with impaired renal function. This can represent an important advancement, taking into consideration that nephropathy is a frequent severe chronic complication of DM, and the administration of selective SGLT-2 inhibitors can produce only poor therapeutic effects in the glycemic control of these patients.^125,128^

Moreover, sotagliflozin showed a favorable safety profile, indicating that the partial inhibition of intestinal SGLT-1 is not capable of causing the GGM syndrome which is observed in SGLT-1 KO mice when fed with glucose as well as in genetically SGLT-1-lacking humans.^127,129−132^

The combination of sotagliflozin with DPP-4 inhibitors, such as sitagliptin, produced synergistic effects, by increasing the glucose-induced GLP-1 release, in both preclinical and clinical trials. Although further clinical data are expected, these findings were shown to be promising as a new opportunity for the treatment of T2DM patients, in addition to the combinations of SGLT-2 inhibitors and DPP-4 inhibitors (saxagliptin-dapagliflozin and linagliptin-empagliflozin) already approved for T2DM treatment.^125,130,133^

Currently, phase III clinical trials of sotagliflozin in T2DM patients are ongoing, whereas this drug was recently approved by EMA (2019) in combination with insulin therapy in adults with T1DM and body mass index of at least 27 kg/m^2^, when insulin on its own does not achieve adequate glycemic control. Sotagliflozin is the first oral antidiabetic drug approved for T1DM in Europe, and it was assessed that, in overweight and obese adult T1DM patients, further effects elicited by the administration of sotagliflozin combined with insulin, such as reduction of body weight and blood pressure, can provide greater benefits than risks; in these patients, severe episodes of hypoglycaemia or diabetic ketoacidosis were not observed.^125,131^ However, FDA so far has refused to authorize sotagliflozin in the United States, as an adjunct agent for T1DM, justifying this choice with concerns regarding the possible increase in the prevalence of diabetic ketoacidosis. It was suggested that this risk could be minimized by appropriate insulin dose adjustments, careful patient selection, and monitoring.^134−136^

**Figure 14:**
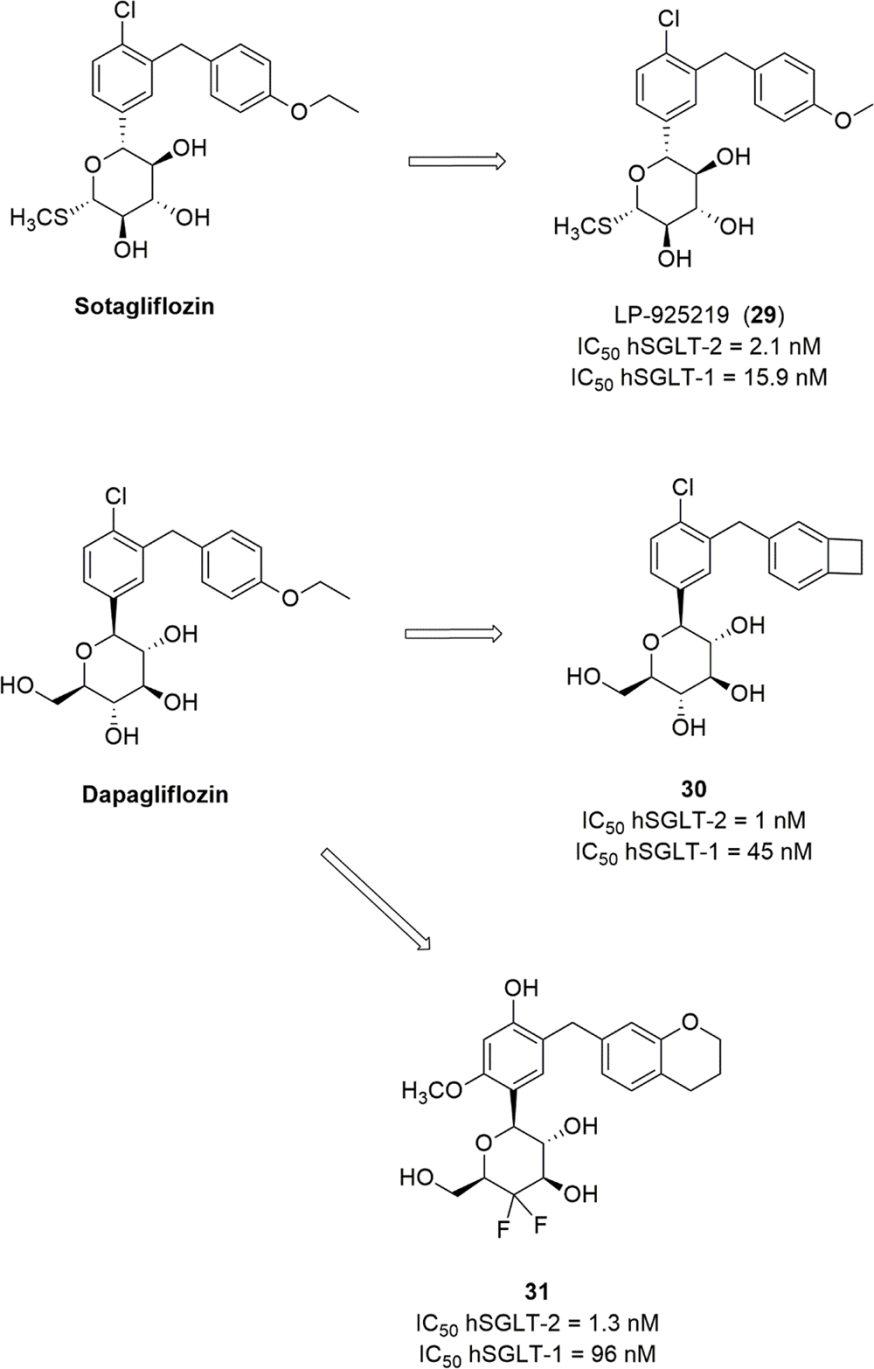
Development of dual SGLT-1/SGLT-2 inhibitors.

The studies carried out with sotagliflozin clearly showed that the simultaneous partial inhibition of both intestinal SGLT-1 and renal SGLT-2, along with the consequently increased incretin release, could provide an optimized control of glycemic homeostasis through a multifactorial mechanism of action, without inducing severe side effects, and thus prompted the continuation of this research.

l-Xyloside LP-925219 (**29**, Figure 14), a close analogue of sotagliflozin, exhibited potent inhibitory activity toward both SGLT subtypes (IC_50_ SGLT-2 = 2.1 nM; IC_50_ SGLT-1 = 15.9 nM); the replacement of the ethoxy group of sotagliflozin with a methoxy in LP-925219 led to lower selectivity toward SGLT-2 over SGLT-1 (IC_50_ hSGLT-1/IC_50_ hSGLT-2 ratio decreased from 20 to 7.6).^123^ LP-925219 showed excellent oral availability (87%) and a relatively long half-life (7 h); its oral administration in rodent models provided both a significant increase of UGE values and reduction of postprandial glycaemia, with increased cecal glucose content and higher plasma GLP-1 levels, thus proving that its anti-hyperglycemic effect derived from inhibition of both intestinal SGLT-1 and renal SGLT-2.^123^

Dapagliflozin-derived benzocyclobutane *C*-glycosides provided further examples of dual SGLT-1/SGLT-2 inhibitors; among them, compound **30** (Figure 14) was selected for its excellent pharmacokinetic profile in several animal models and showed a strong and prolonged anti-hyperglycemic activity in diabetic rodents at 10 mg/kg dose.^137^ More recently, on the basis of the hypothesis that difluoro-substitution in position 5 of the sugar ring could be favorable, 2-aryl-5,5-difluoro-6-(hydroxymethyl)tetrahydro-2*H*-pyran-3,4-diols were synthesized; among them, compounds with balanced SGLT-1/SGLT-2 inhibitory activities (i.e., IC_50_ toward hSGLT-1 ranging from 10 nM to 100 nM and IC_50_ toward hSGLT-2 lower than 10 nM) were selected for pharmacokinetic studies in rats. Out of this series, compound **31** (Figure 14) emerged as a potent SGLT-1/SGLT-2 inhibitor, with a 74-fold preference for SGLT-2 and good safety and pharmacokinetic profiles; it provided good control of postprandial glycemic levels in both SD rats and db/db mice after oral administration at a 10 mg/kg dose.^138^

### 3.5 Intestinal SGLT-1 Inhibitors

The promising multitarget mechanism of action of sotagliflozin prompted the search for new inhibitors targeted to intestinal SGLT-1. It is worth highlighting that increased levels of both mRNA and expression of SGLT-1 were detected in the small intestine of both diabetic animals and humans, determining the increased capacity to absorb glucose and rapid increment of postprandial glycaemia.^139^ In addition, compared to α-glucosidase inhibitors, such as acarbose, which inhibit the production of monosaccharides from the hydrolysis of oligosaccharides in the intestinal lumen, SGLT-1 inhibitors exhibit the advantage to block the absorption of free glucose already present in food not only that originating from the digestion of carbohydrates.

The design of safer SGLT inhibitors appeared to be crucial for the further development of this class of drugs, and, in this view, the preferential inhibition of intestinal SGLT-1 could be a promising strategy. Intestinal SGLT-1 inhibitors not only are lacking of glucosuria-related side effects, such as urogenital infections, but can also provide a mechanism of glycemic control without involving kidney function; this latter feature could be very useful for the treatment of DM in patients suffering from nephropathy.^14^ Interestingly, the partial inhibition of SGLT-1 achieved with sotagliflozin also suggested that a therapeutic window for SGLT-1 inhibition exists, which allows the improvement of glycemic homeostasis without bringing about serious undesired effects that are caused by the total loss of intestinal SGLT-1 activity.

Since the design of highly selective SGLT-1 inhibitors proved to be a challenging task, a strategy to circumvent this difficulty can be the development of agents endowed with low oral bioavailability. Low-adsorbable inhibitors should possess structural and physicochemical properties that can prevent or minimize their systemic absorption after oral administration and, thus, allow them to act exclusively on the SGLT-1 subtype expressed in the gastrointestinal tract.

**Figure 15:**
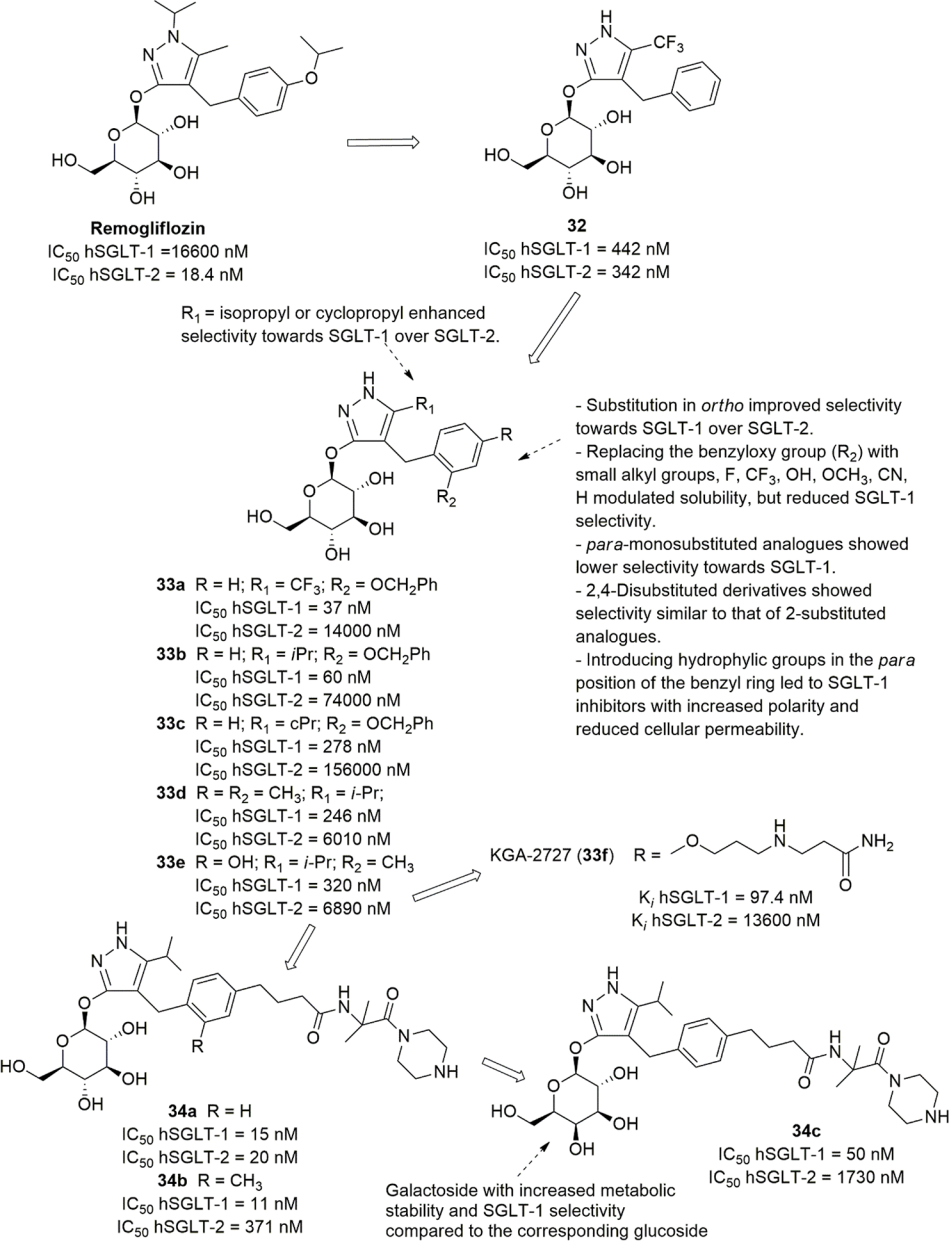
4-Benzyl-1*H*-pyrazol-3-yl β-d-glycopyranosides endowed with selective hSGLT-1 inhibitory activity.

Initial studies aimed at identifying selective SGLT-1 inhibitors started from the design of remogliflozin analogues. In the course of this research, 4-benzyl-5-trifluoromethyl-1*H*-pyrazol-3-yl β-d-glucopyranoside (**32**, Figure 15) was identified as an interesting dual SGLT-1/SGLT-2 inhibitor, with comparable IC_50_ values, and was assumed as a lead compound for the design of analogues with improved selective inhibitory activity against SGLT-1 over SGLT-2.^140^ The substituent in the position 5 of the pyrazole ring as well as the substitution pattern of the benzyl moiety was shown to be crucial to modulate selectivity and potency toward the two SGLT subtypes. In particular, the selectivity toward SGLT-1 over SGLT-2 was enhanced by (a) the replacement of the trifluoromethyl group in position 5 (such as in compounds **32** and **33a**, Figure 15) with an *i*-propyl or cyclopropyl group (R_1_) (such as in compounds **33b**–**e**, Figure 15) and (b) the introduction of a substituent in the *ortho* position of the benzyl ring (R_2_), in particular, a benzyloxy group. The most selective SGLT-1 inhibitor of this series was compound **33b**, endowed with 1200-fold selectivity toward hSGLT-1 over hSGLT-2 (IC_50_ hSGLT-1 = 60 nM; IC_50_ hSGLT-2 = 74000 nM). It was found to be scarcely effective in reducing postprandial glycaemia in rats, and the authors suggested that this unsatisfying result might be attributable to low hydrosolubility of the compound, which makes it unable to compete with glucose in the interaction with intestinal SGLT-1.

To improve the pharmacokinetic profile, the *o*-benzyloxy group (R_2_) was replaced by other less hydrophobic substituents, such as F, CF_3_, OH, CN, or small alkyl groups, which in any case led to lower selectivity for SGLT-1 compared to **33b** (e.g., **33d**, **33e**, Figure 15). The displacement of the substituent from the *ortho* to the *para* position of the benzyl ring also caused a significant decrease in SGLT-1 selectivity. However, compounds **33d** and **33e** (Figure 15), which bear a 2,4-disubstituted benzyl ring, were identified as good SGLT inhibitors, with 24-fold and 22-fold selectivity toward SGLT-1 over SGLT-2, respectively, along with better solubility and metabolic intestinal stability than parent **33b**.^140^

The results of a preclinical study, carried out in streptozotocin (STZ)-nicotinamide-induced diabetic rats, evidenced the significant effectiveness of compounds **33d** and **33e** in reducing glycemic levels in an oral carbohydrate tolerance test, without inducing UGE increase, and, therefore, strongly suggested that this activity was the consequence of the inhibition of intestinal SGLT-1 rather than of renal SGLTs.^140^

Compound KGA-2727 (**33f**, Figure 15), an analogue of **33e**, showed 140-fold selectivity for SGLT-1 over SGLT-2. This glucoside significantly inhibited the absorption of glucose in rat small intestine, in a dose-dependent manner, thus controlling the increase of glycemic levels after glucose loading; similarly to parent *O*-glucosides **33d** and **33e**, it did not induce any increase in UGE values. In addition, **33f** significantly increased the plasma level of GLP-1, which reasonably was responsible for the observed reduction of food intake in Zucker diabetic fatty rats.^141^ Chronic treatment of these animals with **33f** was also capable of preventing the development of both pancreatic β-cell and kidney dysfunctions, which are typical long-term alterations induced by hyperglycemia (especially postprandial hyperglycemia) in the progression of DM.^141^

Interestingly, at the doses used in the above-mentioned studies, none of the tested pyrazole *O*-glucosides caused abdominal adverse effects consequent to intestinal SGLT-1 inhibition.^140,141^

Although pharmacokinetic studies on some of these SGLT-1 selective inhibitors evidenced low systemic exposure, inactive aglycones produced by the hydrolytic activity of intestinal β-glucosidases were detected in the plasma, such as in the case of compounds **33a** and **33e**,^140^ which might be potentially responsible for systemic undesired effects. Therefore, appropriate structural modifications were designed to improve the hydrophilicity of SGLT-1 inhibitors and thus to reduce cellular permeability and systemic absorption of both glucosides and their aglycones.

With this aim, Fushimi and colleagues continued their research by synthesizing a series of 4-benzyl-5-isopropyl-1*H*-pyrazol-3-yl β-d-glycosides bearing novel hydrophilic moieties on the benzyl ring.^13^ A butanamide chain was shown to be a favorable moiety to inhibit SGLT-1, and, subsequently, polar substituents, such as hydroxyalkyl or amide groups, were introduced on the amide nitrogen. *O*-Galactoside derivative **34c** (Figure 15) exhibited an interesting 35-fold selectivity for SGLT-1 over SGLT-2 (IC_50_ hSGLT-1 = 50 nM; IC_50_ hSGLT-2 = 1730 nM); in addition, both galactoside **34c** and its aglycone showed low cellular permeability in a Caco-2 cell permeability test.^13^ Moreover, in STZ-induced-diabetic rats, **34c** reduced plasma glucose levels after oral loading of glucose or sucrose, in a dose-dependent manner, showing greater efficacy than acarbose.^13^ Instead, the corresponding glucoside **34a** (Figure 15) showed scarce selectivity toward the two SGLT subtypes; the introduction of a methyl group in position 2 of the benzyl ring of **34a** provided a more selective SGLT-1 inhibitor, **34b** (Figure 15), which, however, showed lower metabolic stability and anti-hyperglycemic capability than those of galactoside **34c**.^13^

**Figure 16:**
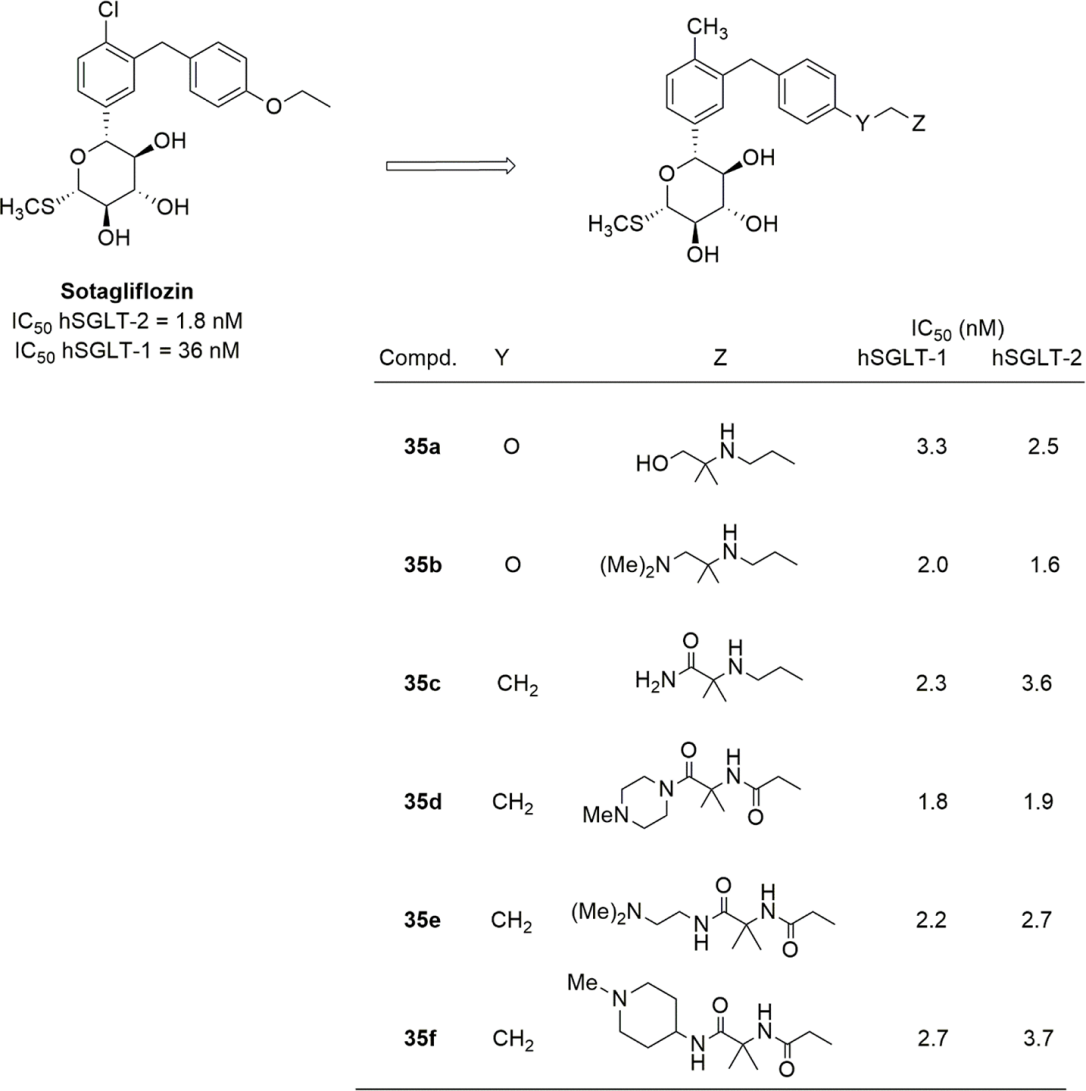
Sotagliflozin-derived low adsorbable dual SGLT-1/SGLT-2 inhibitors.

Subsequent studies in this field pursued the main objective to identify new low adsorbable SGLT inhibitors. Goodwin and colleagues reported a design strategy, which, starting from sotagliflozin, allowed them to synthesize a series of novel *C*-xyloside derivatives.^14^ Regarding both SGLT inhibitory potency and oral bioavailability of the novel derivatives, the distal portion of the substituent in the *para* position of the benzyl ring (Z, Figure 16) was shown to be the most critical moiety, whereas the substituent in the position 4 of the proximal phenyl ring (CH_3_, Cl) or the linker Y (O, CH_2_) did not appear to exert a marked influence. In the portion Z, different functional groups were introduced, such as hydroxyl and differently functionalized amines or amides. In particular, the presence of basic amine groups, which can be prevalently ionized under physiological conditions, provided the most successful results; in fact, compounds **35a**–**d** (Figure 16) exhibited potent inhibitory effectiveness toward both SGLT-1 and SGLT-2, along with a very scarce oral bioavailability. However, the observed instability of some of them in the synthetic conditions limited their investigation and stimulated further modifications of the portion Z, leading to secondary or uncyclized tertiary amides, such as **35e** and **35f** (Figure 16), which were shown to be more stable. Out of these latter compounds, **35e** (LX2761, Figure 16) was selected as a preclinical candidate on the basis of pharmacokinetic and pharmacodynamic data.^14^

In an oral glucose tolerance test performed in both diabetic and nondiabetic animals, the oral administration of compound **35e** determined an increase of cecal glucose amount, due to reduced SGLT-1-mediated absorption; as a consequence, a significant reduction of glycemia and an increase of postprandial plasmatic GLP-1 levels were detected. In contrast with the significant glucosuria increment determined by the parent sotagliflozin, a very scarce effect on UGE was observed after oral administration of **35e**, thus demonstrating that the action of this novel xyloside is restricted to the intestinal SGLT-1 subtype, despite its capability to inhibit both SGLT-1 and SGLT-2 with similar potency.^14,142^ In addition, appropriate doses of **35e** were assessed that brought about glucose-lowering effectiveness without causing intestinal adverse effects.^142^

**Figure 17:**
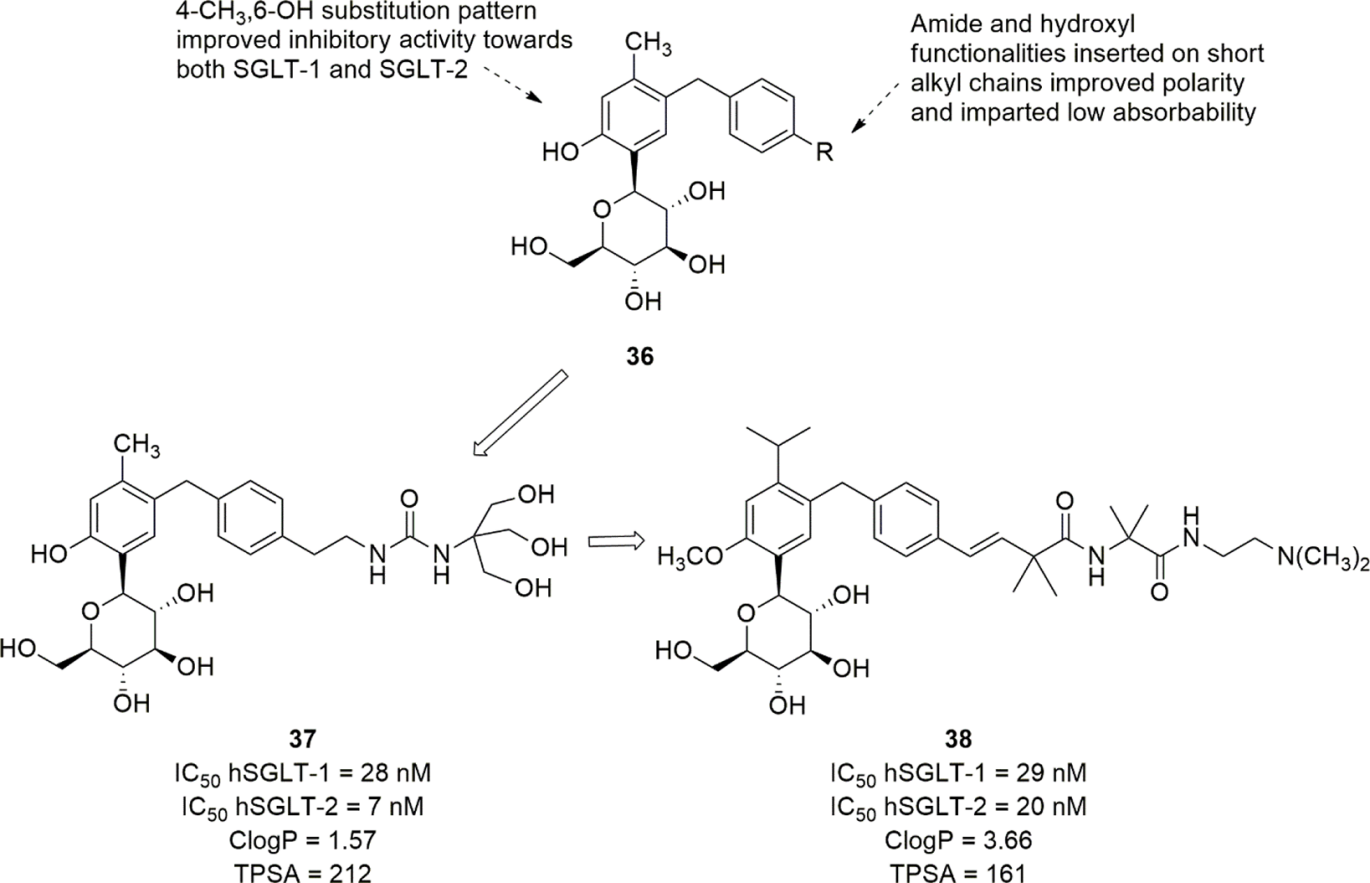
Design of new low adsorbable dual SGLT-1/SGLT-2 inhibitors.

A similar design strategy was reported by Kuroda et al. for the synthesis of new *C*-glycoside derivatives, endowed with increased TPSA and low oral bioavailability.^143^ Starting from the 2-(5-benzyl-2-hydroxy-4-methylphenyl)-6-(hydroxymethyl)tetrahydro-2*H*-pyran-3,4,5-triol scaffold (**36**, Figure 17), highly polar functional groups were introduced in the *para* position of the benzyl ring; in particular, an amide group or urea moiety was linked to the aromatic ring through an ethyl or propyl chain, whereas hydroxyl or amide groups were introduced at the distal tail of these substituents, thus increasing both TPSA and the number of H-bond acceptor/donor groups. The substitution pattern on the proximal benzene ring, i.e., a methyl group in position 4 and an hydroxyl group in position 6, was kept unchanged in most of the tested compounds (Figure 17) since it proved to be beneficial to improve inhibitory potency against both SGLT subtypes.^143^ Among all synthesized *C*-glucosides, compound **37** (Figure 17) stood out for its significant in vitro inhibitory activity toward both SGLT-1 and SGLT-2 symporters (IC_50_ hSGLT-1 = 28 nM; IC_50_ hSGLT-2 = 7 nM), accompanied by a very low oral bioavailability, which was related to a high TPSA value (212 Å^2^) and a noticeable number (22) of H-bond acceptor/donor groups. Once again, the dose-dependent glucose-lowering activity of compound **37** was exclusively ascribable to the inhibition of intestinal SGLT-1, without any significant effect on UGE, at doses of 0.1 and 0.3 mg/kg/twice a day in rats.^143^

However, since it was observed that small amounts of compound **37** can be absorbed in the intestine and accumulated in kidneys for many hours (kidney elimination half-time = 160 h) before being excreted in the urine, the same authors pursued the aim of promoting biliary excretion, to avoid the unwanted effects derived from renal SGLT-2 inhibition. Therefore, new analogues were designed by increasing lipophilicity and, at the same time, maintaining the low absorbability of lead compound **37**. These efforts led to the discovery of a further candidate, SGL5213 (**38**, Figure 17), which exhibited similar IC_50_ values toward both hSGLT-1 and hSGLT-2, along with low membrane permeability and low oral bioavailability.

When orally administered at the dose of 0.3 mg/kg before sucrose loading in SD rats, compound **38** showed a significant glucose-lowering activity, without unwanted gastrointestinal effects. As expected, compound **38** was shown to be mainly excreted via a biliary pathway after intravenous administration in rats. On the basis of the available experimental data, a correlation between lipophilicity (ClogP values >3.5) and biliary excretion was established; at the same time, a value of TPSA of at least 160 Å^2^ was considered necessary to maintain low intestinal absorbability.^15^

### 3.6 Indole-Substituted N- and C-Glycosides

**Figure 18:**
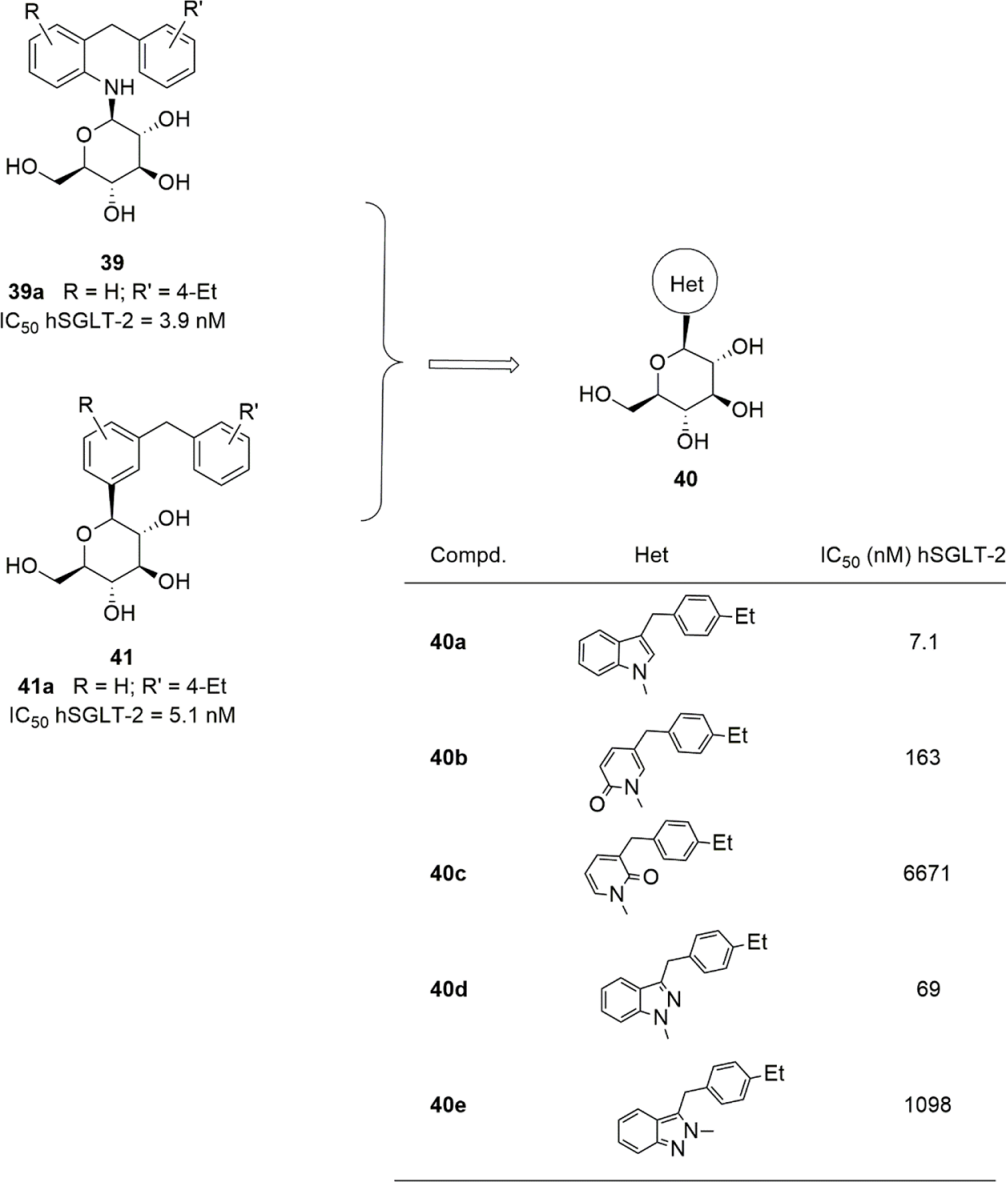
Structures of selected aniline-*N*-glucosides and heteroaromatic-*N*-glucosides.

In the course of research on SGLT-2 inhibitors metabolically more stable than *O*-glycosides, a series of aniline-*N*-glucosides **39** and heteroaromatic-*N*-glucosides **40** (Figure 18) were designed.^144^ Heteroaromatic-*N*-glucosides **40** were the result of the combination of aniline *N*-glucosides **39** and *m*-diarylmethane *C*-glucosides **41**, maintaining a fixed 4-ethylbenzyl moiety.^144^ Among the synthesized aniline-*N*-glucosides, 2-(4-ethylbenzyl)aniline substituted compound **39a** (Figure 18) was shown to be an interesting SGLT-2 inhibitor (IC_50_ hSGLT-2 = 3.9 nM) comparable to the corresponding *C*-glucoside **41a** (IC_50_ hSGLT-2 = 5.1 nM). Despite the interesting SGLT-2 inhibitory activity, the oral administration of compound **39a** in SD rats induced a low value of UGE (93 mg/day) compared to compound **41a** (1485 mg/day). The authors associated this poor in vivo activity to its hydrolytic degradation in an aqueous acid environment, releasing 2-(4-ethylbenzyl)aniline which was isolated in pharmacokinetic studies in rats.

Compounds **40** exhibited a wide range of SGLT-2 inhibitory activity, resulting from weak to appreciable inhibitors. In particular, the derivative containing an indole nucleus (compound **40a**, Figure 18) exhibited an interesting SGLT-2 inhibition value (IC_50_ hSGLT-2 = 7.1 nM) and weak SGLT-1 inhibition (IC_50_ hSGLT-1 = 1956 nM), showing to be a selective SGLT-2 inhibitor (IC_50_ hSGLT-1/IC_50_ hSGLT-2 ratio = 275). It also showed interesting glucosuric effect after oral administration (UGE = 1830 mg/day), similar to that of aryl-*C*-glycoside **41a** (UGE = 1485 mg/day) and 20-fold higher than compound **39a**.^144^

Overall, the comparison of the pharmacokinetic results of compounds **39a** and **40a** indicated that the latter had a lower clearance and better bioavailability, due to higher chemical stability of its *N*-glycoside bond; in fact, its corresponding aglycone was not isolated in pharmacokinetic studies.

Among the derivatives containing the pyridone moiety, 5-(4-ethylbenzyl) substituted compound **40b** (IC_50_ hSGLT-2 = 163 nM, Figure 18) was proven to be about 40-times more active than 3-(4-ethylbenzyl) substituted isomer **40c** (Figure 18, IC_50_ hSGLT-2 = 6671 nM), in which the presence of the carbonyl group in position 2 probably induces an unfavorable spatial arrangement of the 4-ethylbenzyl group in the interaction with the target.^144^ It is worth comparing the inhibitory activity of the 1- and 2-glycosylated benzopyrazole isomers (**40d** and **40e**, respectively, Figure 18) since the 1-glycosylated compound **40d** (IC_50_ hSGLT-2 = 69 nM) was 16 times more active than the 2-glycosylated isomer **40e** (IC_50_ hSGLT-2 = 1098 nM). This result is in agreement with the SARs outlined from the *C*-glycoside analogues in which the relative *meta* position of the sugar group and the distal benzyl was shown to be favorable for the SGLT-2 inhibitory ability.^144^

**Figure 19:**
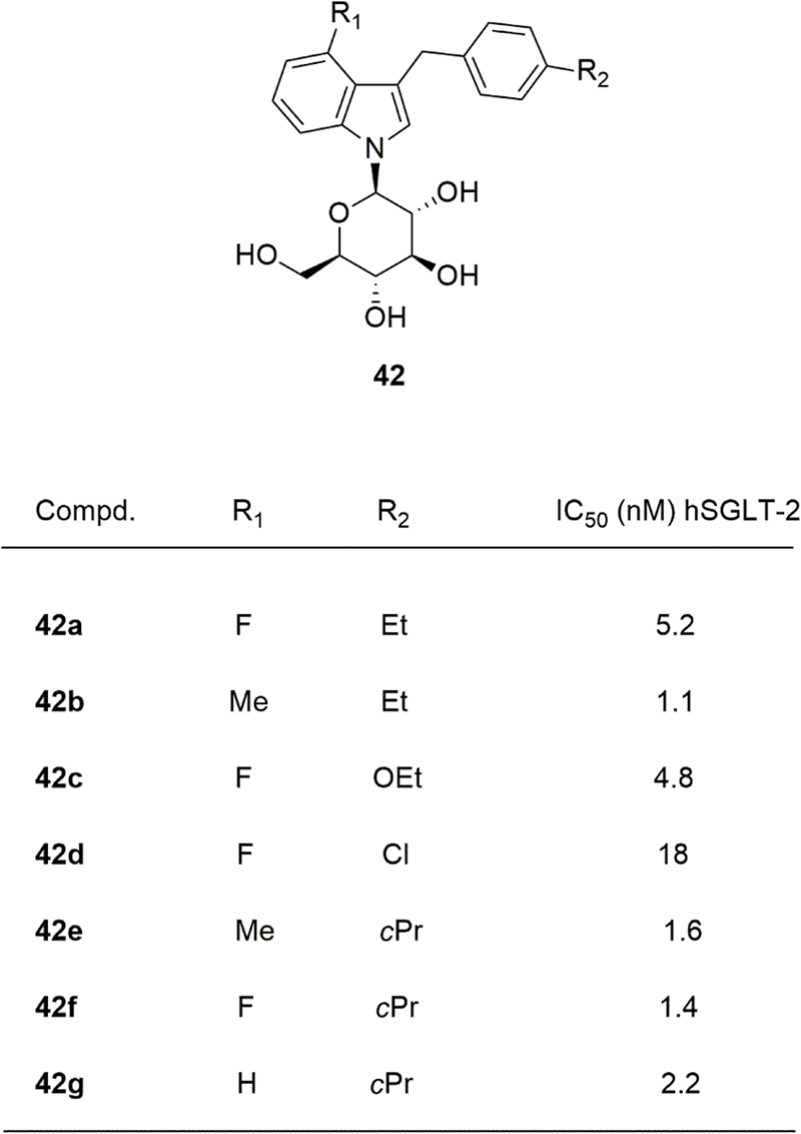
Structures of selected 3-benzylindolyl-*N*-glucosides.

The interesting SGLT-2 inhibitory activity of 3-(4-ethylbenzyl)-1*H*-indole N-glucoside **40a** (Figure 18) led to the development of a new series of differently substituted 3-benzylindole *N*-glucoside derivatives **42** (Figure 19), designed to optimize in vitro and in vivo activity.^145^ First, by keeping fixed the 4-ethylbenzyl group, the effect of both electron-withdrawing and electron-donor groups in 4-position of indole moiety was evaluated. Compounds **42a** and **42b**, 4-F and 4-CH_3_ substituted respectively (Figure 19), showed better inhibitory ability compared with compound **40a**. In particular, compound **42b** appeared to be a better inhibitor (IC_50_ hSGLT-2 = 1.1 nM; UGE = 1664 mg/200 g BW/day) than compound **42a** (IC_50_ hSGLT-2 = 5.2 nM), although the latter induced an increase of the UGE value (2937 mg/200 g BW/day). The replacement of 4-ethyl with different groups, such as ethoxy or chloro, provided compound **42c** (Figure 19, IC_50_ hSGLT-2 = 4.8 nM), which maintained a similar inhibitory activity of precursor **42a**, while 3-(4-chlorobenzyl)-4-fluoro analogue **42d** (Figure 19, IC_50_ hSGLT-2 = 18 nM) proved to be less active.^145^

3-(4-Cyclopropylbenzyl)-4-methyl-1*H*-indole *N*-glucoside (compound **42e**, IC_50_ hSGLT-2 = 1.6 nM), obtained by replacing the ethyl group of **42b** with a cyclopropyl, showed the same SGLT-2 inhibitory activity compared to the parent compound, while the UGE value significantly improved (2830 mg/200 g BW/day). Subsequently, homologous cycloalkyl groups (cyclopentyl and cyclobutyl) were introduced in the *para* position of the benzyl moiety, showing that the increased size of the cycloalkyl ring is unfavorable for the inhibitory activity.

Overall, the best inhibitors appeared to be the 4-cyclopropylbenzyl substituted derivatives (**42e**, IC_50_ hSGLT-2 = 1.6 nM; **42f**, IC_50_ hSGLT-2 = 1.4 nM; **42g**, IC_50_ hSGLT-2 = 2.2 nM); however, the authors underlined that 4-indole unsubstitued compound **42g** proved to be chemically unstable.^145^ Compound **42f** was the most selective toward SGLT-2 over SGLT-1 (IC_50_ hSGLT-1 = 230 nM; IC_50_ hSGLT-1/IC_50_ hSGLT-2 = 164.3). Moreover, these compounds were shown not to inhibit GLUT-1 activity at 10 μM concentration in L6 myoblast cells.^145^ Among all tested compounds, **42a**, **42e**, and **42f** provided extensive UGE in SD rats. Because of its ability to selectively inhibit the SGLT-2 subtype, compound **42f** (TA-1887) was selected for further evaluation as a preclinical candidate. Pharmacokinetic studies indicated that compound **42f** was stable in the presence of human and animal intestinal microsomes in vitro, suggesting that indole-*N*-glucosides are metabolically stable to intestinal β-glucosidase hydrolysis, similarly to the corresponding *C*-glucosides. Moreover, compound **42f** proved to be effective in controlling hyperglycemia in high-fat diet-fed KK mice.^145^

**Figure 20:**
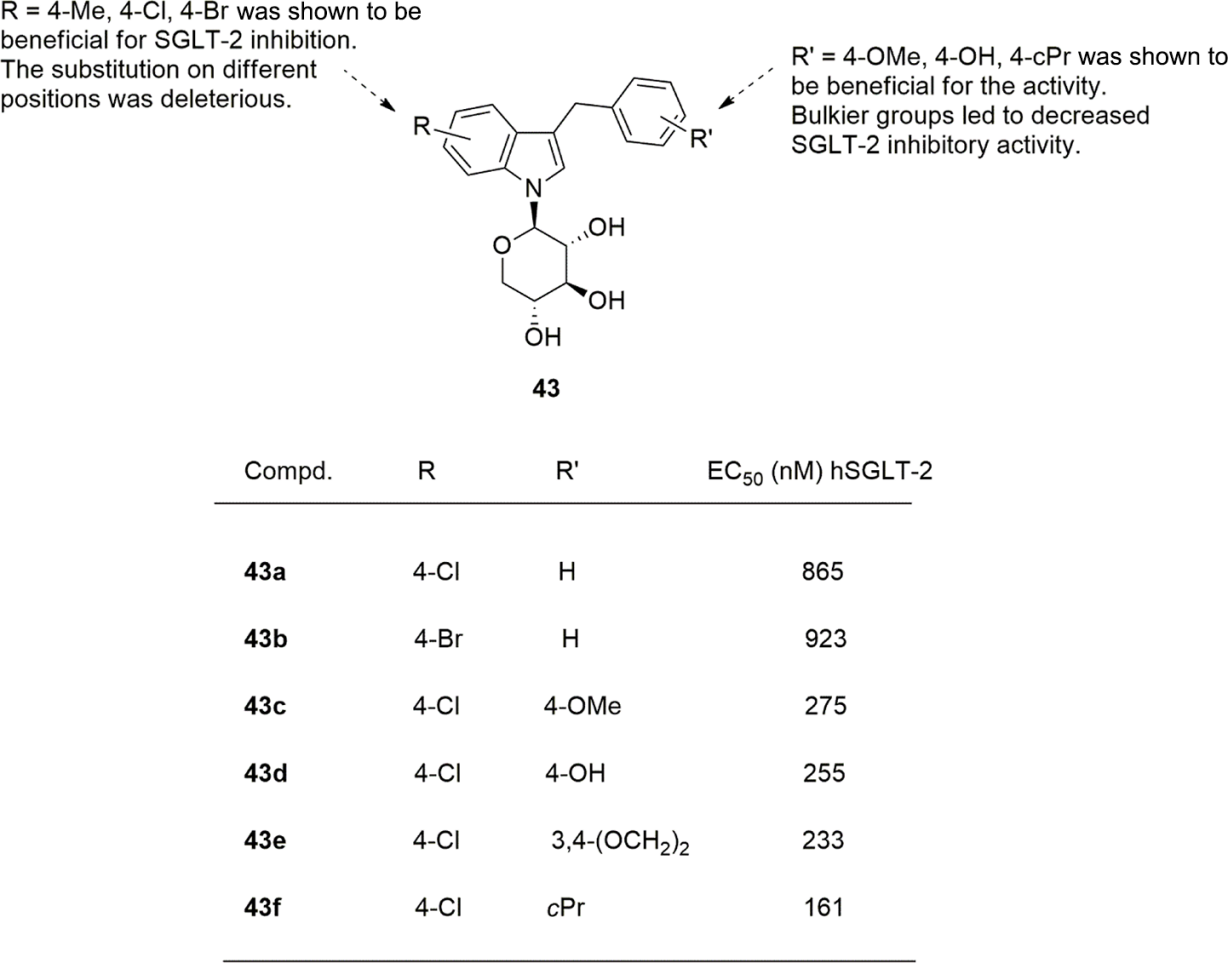
Structures of selected N-linked β-d-xylosides.

In pursuing efforts to identify potent and selective SGLT-2 inhibitors, a new class of 3-benzylindole *N*-xylosides was designed to obtain new derivatives endowed with greater metabolic stability as well as effectiveness compared to *C*-glucoside analogues.^146^ In this context, the synthesis and SARs of a numerous series of *N*-linked β-d-xylosides were reported. 3-Benzyl substituted indole was the scaffold selected as aglycone, whereas d-glucose, a common sugar among SGLT-2 inhibitors, was replaced with d-xylose which maintains the same configuration at C-2, C-3, and C-4 (compounds **43**, Figure 20).^146^ The replacement of d-glucose with d-xylose allowed the authors to predict greater stability to intestinal β-glucosidases and therefore better oral bioavailability. Moreover, a series of SGLT-2 inhibitors obtained by replacing d-glucose with l-xylose provided potent *C*-xyloside inhibitors both in vitro and in vivo, leading to the development of sotagliflozin.^120^ The introduction of substituents endowed with different electronic properties on the benzene ring of indole provided some interesting inhibitors, and above all 4-chloro (**43a**, EC_50_ hSGLT-2 = 865 nM) and 4-bromo (**43b**, EC_50_ hSGLT-2 = 923 nM) substituted indoles (Figure 20) were more favorable than the 4-methyl substituted analogue. These substituted indole derivatives were shown to be from 6.5-fold to 8.2-fold more effective compared to the unsubstituted analogue. In any case, 4-substituted indole derivatives were found to be favorable for SGLT-2 inhibition compared to 5-, 6-, or 7-substituted isomers.^146^

Starting from 4-chloroindolyl analogue **43a**, changes directed to the benzyl ring were designed by introducing substituents endowed with different electronic natures in various positions. The introduction of a 4-methoxybenzyl group (**43c**, EC_50_ hSGLT-2 = 275 nM, Figure 20) produced interesting activity compared to unsubstituted analogue **43a**, whereas its displacement to both 2- and 3-positions turned out to be detrimental. Moreover, the insertion of bulkier alkyl/alkyloxy/aryl groups in the 4-position of the benzyl moiety as well as the introduction of different substituents (such as 2,4-diOCH_3_ or 3F,4-OCH_3_) induced a decrease of activity, attributable to the steric hindrance induced by the benzyl portion in the target interaction.^146^

The 4-hydroxybenzyl-substituted derivate **43d** (EC_50_ hSGLT-2 = 255 nM) corroborates this hypothesis. The replacement of the 4-methoxybenzyl group with 4-fluorobenzyl or benzofused rings was deleterious for the SGLT-2 inhibitory activity, except for the 3-[(2,3-dihydrobenzo[b][1,4]dioxin-6-yl)methyl]-1*H*-indolyl-substituted derivative (**43e** EC_50_ hSGLT-2 = 233 nM) which showed activity comparable to **43c**. In most cases, the EC_50_ values significantly increased, except for the 4-cyclopropylbenzyl-substituted analogue (**43f**, EC_50_ hSGLT-2 = 161 nM, Figure 20), which was shown the most effective of the series toward hSGLT-2. Selected compounds endowed with better hSGLT-2 inhibition showed no significant selectivity for hSGLT-2 versus hSGLT-1 (EC_50_ hSGLT-1/EC_50_ hSGLT-2 = 0.8–2.1). The best in vitro inhibitor 4-chloro-3-(4-cyclopropylbenzyl)-1*H*-indole *N*-xyloside (**43f**) proved to be metabolically stable with a low clearance and good oral bioavailability in SD rats. Moreover, it was shown to increase UGE and urine volume from 12-fold to 783-fold, at different doses in rats. Oral administration of **43f** at a 10 mg/kg dose in STZ-induced diabetic rats was shown to reduce blood glucose levels.^146^

**Figure 21:**
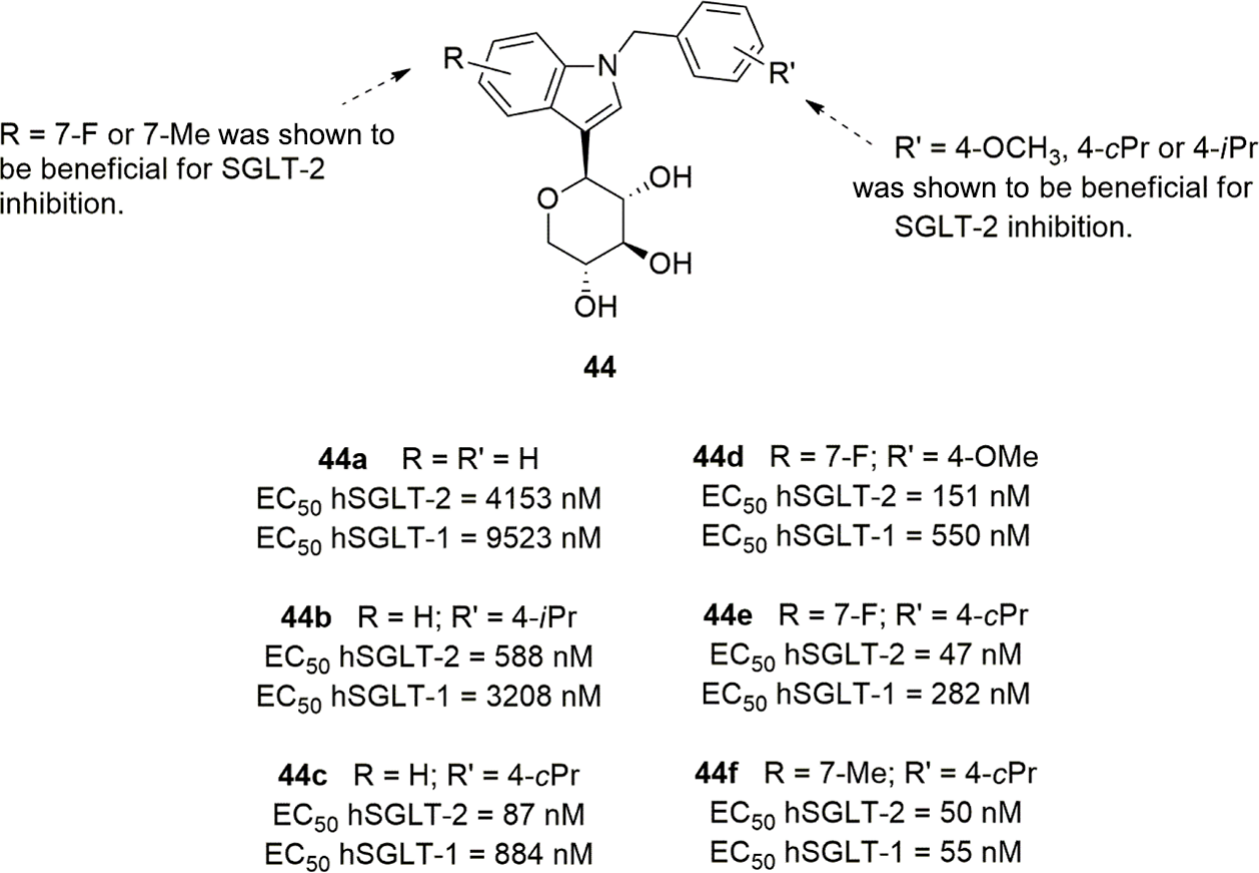
Structures of selected *C*-indolylxylosides.

In continuing this research, a new series of differently substituted 1-benzyl-3-(β-d-xylopyranosyl)-1*H*-indole analogues (compounds **44**, Figure 21) were reported.^147^ Substituents of different chemical natures were introduced in the *para* position of the benzyl portion, while the xylose was kept constant in position 3 of the indole nucleus.^147^ By assuming the unsubstituted derivative **44a** (Figure 21) as a reference compound, it emerged that the insertion of a fluorine atom in the *para* position of the *N*-benzyl group was deleterious for the activity, while its replacement with a methyoxy group improved the inhibitory activity. The presence in the same position of a weak electron-donor group produced different effects, which was shown to be significantly influenced by steric hindrance; in fact, the *n*-propyl substituted derivative (**44b**, EC_50_ hSGLT-2 = 588 nM) was about 6-fold more active than the *t*-butyl substituted analogue; moreover, the 4-cyclopropyl-substituted analogue showed a more marked SGLT-2 inhibitory activity (**44c**, EC_50_ hSGLT-2 = 87 nM).^147^

Changes targeting the indole moiety were suggested by previously acquired SARs for both N/C-indolyl glycosides.^146^ The introduction of a fluorine atom in position 4 or in position 7 of the 4-methoxybenzylindole derivative produced opposite effects; in fact, the 7-fluoro-substituted compound **44d** (EC_50_ hSGLT-2 = 151 nM) was 5-fold more active than the unsubstituted parent and at least 6-fold more active than the 4-F substituted isomer. Furthermore, the introduction in position 7 of the 4-cyclopropylbenzyl-substituted derivative **44c** of an electron-withdrawing group, such as F (**44e**, EC_50_ hSGLT-2 = 47 nM), or an electron-donating group, such as methyl (**44f**, EC_50_ hSGLT-2 = 50 nM), provided the most active compounds of the series. Lastly, the authors underlined that *C*-indolylxyloside derivatives were generally more active than *N*-indolylxyloside analogues, and, moreover, the substituent on C-6 position of the sugar moiety plays a critical role in the SGLT-2 inhibitory ability.^146,147^

Similarly to *N*-indolylxylosides **43**, none of tested C-indolylxylosides **44** showed significant selectivity for SGLT-2 (EC_50_ hSGLT-1/EC_50_ hSGLT-2 ratio = 0.8–10.2). Compound **44e** was selected for further pharmacokinetic studies in rats, from which favorable properties emerged. Moreover, it exhibited an anti-hyperglycemic effect in STZ-diabetic SD rats, lowering the blood glucose level of 37% at the dose 20 mg/kg.^147^

**Figure 22:**
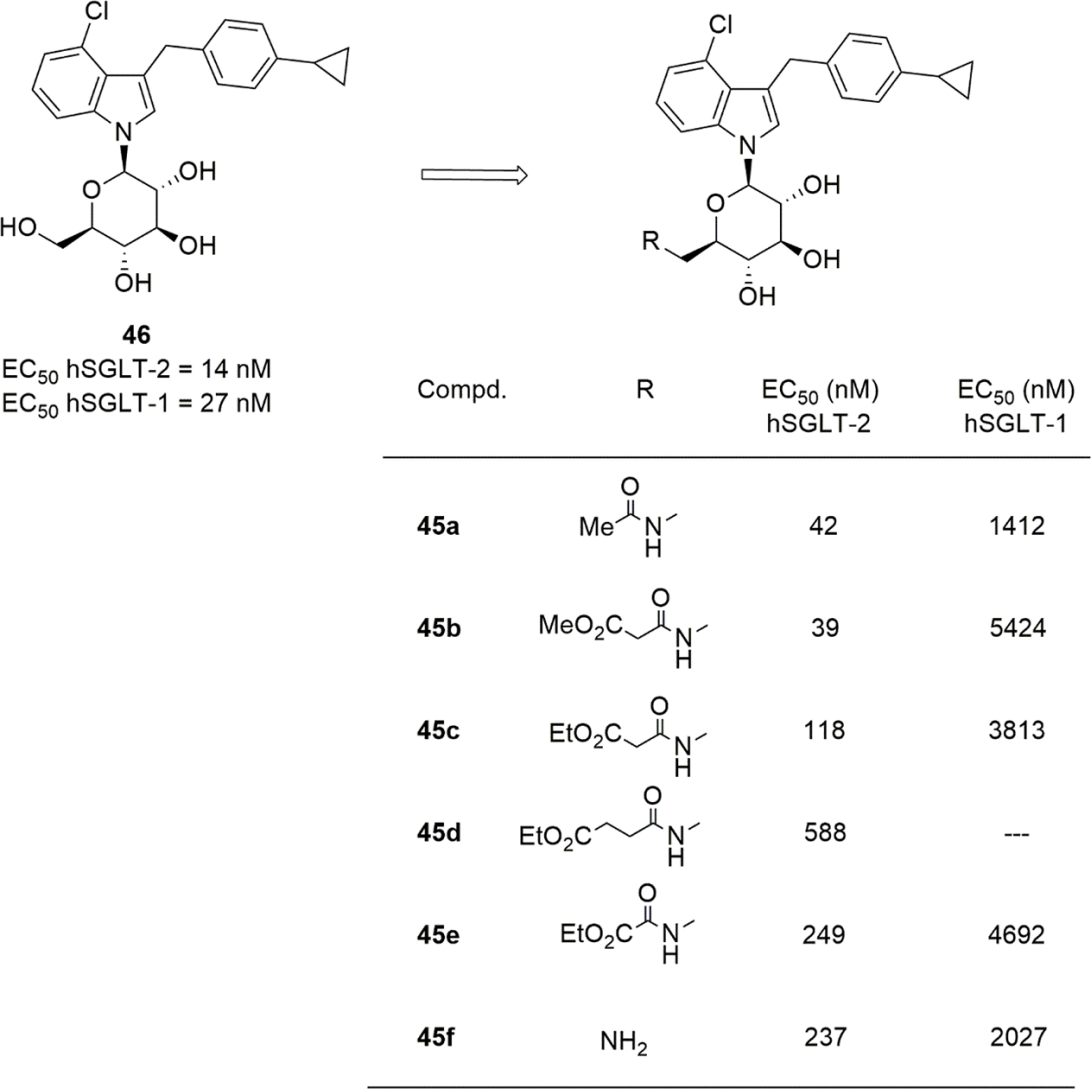
Selected 3-(4-cyclopropylbenzyl)-1*H*-indole *N*-glucosides.

Overall, the studies reported by Yao et al. suggested that the aglycone 4-chloro-3-(4-cyclopropylbenzyl)-1*H*-indole proved to be the most favorable moiety for the SGLT-2 inhibitory ability of *N*-glycosides.^146^ In the pursuit of this research, Chu et al. considered appropriate to assess the effects of changes in the sugar C-6 position of *N*-glycosides, the aglycone 4-chloro-3-(4-cyclopropylbenzyl)-1*H*-indole being fixed.^148^ A wide series of compounds was reported differing in the substitution in the position 6 of the sugar moiety; among them, 1-[6-(acetylamino)-6-deoxy-β-d-glucopyranosyl]-4-chloro-3-(4-cyclopropylbenzyl)-1*H*-indole (**45a**, EC_50_ hSGLT-2 = 42 nM), and the corresponding 6-[(3-methoxy-3-oxopropanoyl)amino] substituted (**45b**, EC_50_ hSGLT-2 = 39 nM) showed the best inhibitory ability against hSGLT-2. In addition, **45b** showed the best selectivity for hSGLT-2 versus hSGLT-1 (EC_50_ hSGLT-1/EC_50_ hSGLT-2 = 139), and both compounds **45a** and **45b** were found to be more selective SGLT-2 inhibitors than the previously reported *N*-indolylglucoside **46** (Figure 22, EC_50_ hSGLT-2 = 14 nM; EC_50_ hSGLT-1/EC_50_ hSGLT-2 ratio = 2). Both compounds belong to 6-amido derivatives and appeared to be better inhibitors than amino unsubstituted analogue **45f** (EC_50_ hSGLT-2 = 237 nM, Figure 22).^148^ The introduction of electron-withdrawing groups or the elongation of the chain to chloroethyl or bromoethyl was unfavorable as well as the presence of bulky moiety such as isopropyl, cyclohexyl, aryl, or heteroaryl groups on the 6-acetylamino head. The replacement of 6-[(3-methoxy-3-oxopropanoyl)amino] group (compound **45b**) with 6-[(3-ethoxy-3-oxopropanoyl)amino] group (compound **45c** EC_50_ hSGLT-2 = 118 nM) reduced SGLT-2 inhibitory ability. By comparing the inhibitory ability of carboxyethyl-substituted compounds **45c**–**e**, the (3-ethoxy-3-oxopropanoyl)amino group in position 6 (**45c**) was the most favorable, while the insertion or the removal of a methylene group (**45d** EC_50_ hSGLT-2 = 588 nM; **45e** EC_50_ hSGLT-2 = 249 nM, respectively) induced a clear reduction of activity.^148^ Overall, these results indicated that bulky groups on position 6 of the sugar moiety were detrimental.

The introduction of urea and thiourea groups or triazole at the C-6 position of the sugar moiety was generally detrimental for both the activity and selectivity toward hSGLT-2 versus hSGLT-1. Compounds **45a** and **45b** were further studied to evaluate their ability to induce UGE in normal SD rats after oral glucose load; however, both selected compounds showed poor pharmacokinetic properties and resulted in an unfavorable outcome in this glucosuria study in rats.^148^

**Figure 23:**
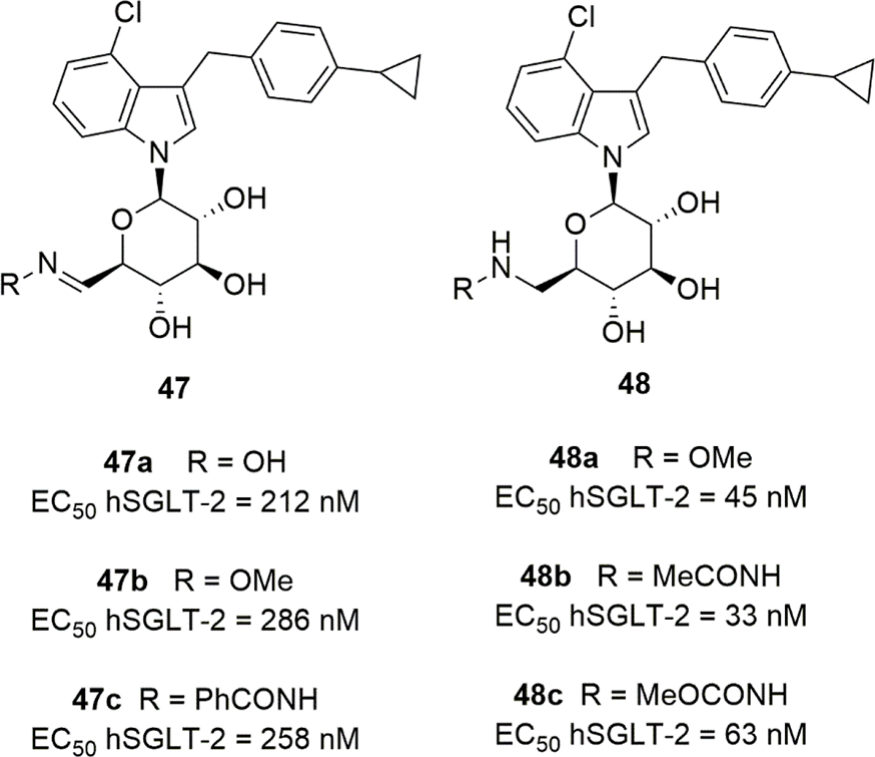
Selected 4-chloro-3-(4-cyclopropylbenzyl)-1*H*-indole *N*-glycosides modified at the C-6 position of the sugar moiety.

Starting from compound **46** (Figure 22),^148^ 6-oxime- and 6-amido-6-deoxyglucose derivatives (**47** and **48**, Figure 23) were synthesized, again keeping constant the aglycone 4-chloro-3-(4-cyclopropylbenzyl)-1*H*-indole.^149^ Among the oxime derivatives, 6-[(hydroxyimino)methyl] substituted compound **47a** (Figure 23) proved to be the best inhibitor (EC_50_ hSGLT-2 = 212 nM), although the corresponding methoxyimino analogue also showed interesting levels of activity (**47b**, EC_50_ hSGLT-2 = 286 nM); however, *C*-glycosyl analogues (such as compound **27**, EC_50_ hSGLT-2 = 46 nM, Figure 12) generally produced better SGLT-2 inhibition levels.^119^

The *N*-acylhydrazone analogues, designed by replacing the oxime ether oxygen with an amido group, generally provided interesting inhibitors, the best of which was phenylhydrazone **47c** (Figure 23, EC_50_ hSGLT-2 = 258 nM). Interestingly, hydroxylamines and hydrazides **48**, obtained by reduction of the corresponding oximes and *N*-acylhydrazones **47**, respectively, provided the best inhibitors (EC_50_ = 33–294 nM) of the series. The derivative bearing a methoxyamine group (**48a** EC_50_ hSGLT-2 = 45 nM) was shown to be the most potent SGLT-2 inhibitor, while the unsubstituted or ethoxyamine substituted analogues reduced the SGLT-2 inhibition by 3.5- and 6.6-fold, respectively.^149^ Among hydrazides, **48b** (EC_50_ hSGLT-2 = 33 nM) showed the highest level of SGLT-2 inhibition similar to the 6-methoxyamino-substituted analogue **48a**, followed by **48c** (EC_50_ hSGLT-2 = 63 nM, Figure 23).

The selectivity and in vivo studies of compound **48b**, selected as the best inhibitor, showed poor selectivity for SGLT-1 (hSGLT-1 EC_50_ = 37 nM). When orally administered in normal SD rats, **48b** was proven to increase UGE only at relatively high dose compared to dapagliflozin.^149^ Overall, these results show that the groups in the C-6 position of the 6-deoxyglucoside moiety play a critical role in SGLT-2 inhibition. In particular, the presence of small size substituents appears favorable as well as groups endowed with higher flexibility, which could be able to better interact with the target.

## 4 Conclusions and Perspectives

The management of T2DM and its complications requires a complex therapeutic approach, generally realized through combinations of drugs with different mechanisms of action. Despite the availability of different classes of antidiabetic drugs, glycemic control in DM still represents a difficult challenge, and, as a result, the hyperglycemia-induced pathologies associated with DM, such as cardiovascular and renal complications, occur with high incidence.

In this context, the recent approval of SGLT-2 inhibitors (gliflozins) was an important novelty, due to the unique characteristics of these therapeutic agents. In particular, it is remarkable that these drugs are capable of not only improving glycemic control without risk of severe hypoglycemia but also of exhibiting significant protective effects on heart and kidneys; these latter features can significantly contribute to counteract the development of DM-associated cardiovascular and renal complications. Interestingly, the mechanism underlying the anti-hyperglycemic activity of gliflozins is totally independent of insulin, and this feature prompted clinical trials also for patients with T1DM, in combination with insulin therapy.

The novel activity profile exhibited by these drugs even gave rise to the question of whether SGLT-2 inhibitors can change the clinical course of DM. In fact, from the results available so far, it appeared that, at an early stage of therapy, a gliflozin associated with metformin and a dipeptidylpeptidase-4 inhibitor could slow the progression of T2DM,^110^ albeit it is necessary to ascertain this possibility in a higher number of newly diagnosed T2DM cases.

Moreover, the cardiorenal benefits produced by the treatment with SGLT-2 inhibitors were shown to be effects of this drug class partly independent of the activity on blood glucose levels and body weight; clinical trials with known gliflozins highlighted additional mechanisms of action that were unexpected at the time of the approval of these drugs, and, consequently, these findings could pave the way for an extension of their therapeutic usefulness. Initially, SGLT-2 inhibitors were approved for the treatment of T2DM, particularly in young and middle-aged patients with obesity or metabolic syndrome; currently, their use has been extended to patients with T2DM associated with cardiovascular or renal pathologies as well as to patients with T1DM. Moreover, considering that the improvement of cardiorenal functions emerged also in nondiabetic subjects, several clinical trials are underway to ascertain whether gliflozins can also be used in nondiabetic patients with heart or kidney failure or, in the case of IGT diagnosis, to prevent the onset of T2DM.^27^

In the last two decades, extensive SAR studies highlighted that the anti-hyperglycemic efficacy as well as pharmacokinetic, selectivity, and safety profiles of gliflozins can be markedly influenced and modulated by defined structural aspects. Two main requisites were found to be critical for SGLT-2 inhibition, i.e., an hydrophobic moiety, preferentially a diarylmethane portion, and a glycoside portion. Moreover, a fundamental feature required to achieve drug-like glycosides and develop them as oral antidiabetic agents is their metabolic stability to intestinal β-glycosidases; especially, *C*-glycosides were shown to be stable to these hydrolases and thus were widely explored to develop SGLT inhibitors as drug candidates. Different substitution patterns can be introduced in the hydrophobic portion, whereas d-glucopyranose generally proved to be the most beneficial glycoside scaffold related to highly potent and selective SGLT-2 inhibition. However, certain modifications of the sugar portion were proven to be tolerated and allowed an extension of the chemical space for SGLT inhibitors, in some cases shifting preferential inhibition toward SGLT-1 subtype and leading to the identification of dual SGLT-1/2 inhibitors (such as sotagliflozin and derivatives **35**, **37**, **38**, Figures 16 and 17).

In the past few years, the investigation concerning dual SGLT-1/2 inhibitors as well as intestinal SGLT-1 inhibitors has attracted growing interest and has suggested further opportunities for developing new antidiabetic drugs. On the whole, the results of these studies allow some interesting considerations. First, the efforts to obtain a higher selectivity toward SGLT-1 over SGLT-2 highlighted that this might be a challenging task since so far a limited number of selective SGLT-1 inhibitors have been reported, and most dual SGLT-1/2 inhibitors showed a preference toward SGLT-2. The SAR studies suggested that effective SGLT-1 inhibition requires more specific or additional structural features, whereas the SGLT-2 site appears to be capable to fit and effectively bind a wider variety of inhibitors. The recently reported models of hSGLT-1 and hSGLT-2 interestingly offered a plausible explanation for the higher potency of many inhibitors toward SGLT-2 over SGLT-1, by evidencing two main possible determinants for subtype selectivity: (a) the presence of additional aromatic residues, in particular, His268 included in the EL5c loop of hSGLT-2, but absent in hSGLT-1, contributes to form an hydrophobic pocket surrounding the central ring of aglycon, by establishing significant additional interactions with inhibitors; (b) the different Na^+^/substrate stoichiometry of SGLT-1 and SGLT-2 subtypes determines an allosteric control of target conformations, favoring in SGLT-2 a partially occluded conformation with enhanced inhibitor affinity.^21^

In remogliflozin-derived dual inhibitors, it was feasible to increase the selectivity ratio toward SGLT-1 by means of appropriate substituents on both pyrazole and benzyl portions; selected compounds (such as **33d**, **33e**, **33f**, **34c**, Figure 15) endowed with interesting SGLT-1/SGLT-2 selectivity ratios were shown to be worth of further preclinical investigations. Sotagliflozin-derived dual inhibitors (such as compounds **35**, Figure 16) showed lower selectivity, being active almost to the same extent against both SGLT subtypes (similarly to glycosides **37**, **38**, Figure 17). Lastly, some poorly selective SGLT inhibitors were identified among *N*-glycosides; these latter offered further examples of metabolically stable SGLT inhibitors, among which promising preclinical candidates (such as compounds **42f**, **43f**, and **44e**, Figures 19–21) were identified, thus suggesting that structural diversity can be pursued in the design of new SGLT inhibitors. Compared to *C*-glycoside analogues, *N*-glycosides were generally less potent and selective SGLT-2 inhibitors, and some of them also provided unsatisfactory glucosuric activity. However, these outcomes, which were unfavorable with regard to glucosuria-related anti-hyperglycemic effects, might be reconsidered from a different prospect; in fact, it might take advantage of both poor SGLT-2/SGLT-1 selectivity and scarce oral bioavailability shown by certain *N*-glycosides to develop new low adsorbable SGLT inhibitors. In this view, further investigation on certain above-mentioned *N*-glycosides (such as selected compounds of series **43**, **44, 48**, Figures 20, 21, and 23) might be desirable to shed light on their potential as lead compounds of a new series of SGLT inhibitors.

The results available so far highlighted that a partial inhibition of intestinal SGLT-1 symporter, associated with SGLT-2 inhibition, can be a useful strategy to achieve a good glycemic control through a multitargeted mechanism of action. We believe that, currently, the most promising approach to achieve new SGLT inhibitors and further develop this class of therapeutic agents could be the design of dual SGLT-1/SGLT-2 inhibitors or polar and low adsorbable compounds selectively directed to intestinal SGLT-1 subtype; the modulation of physicochemical properties emerged as a successful approach to identify drug-like candidates in this field and could be further explored. In fact, considering that the design of SGLT-1 inhibitors represents the most recent phase of this research, it can be expected and desirable that further studies will be carried out in a more varied chemical space to obtain new inhibitors and extend SAR knowledge.

The identification of new SGLT-1 inhibitors could also allow the extension of knowledge of this symporter and its physiological roles. In fact, SGLT-1 is expressed in several tissues and appears to exert diverse functions. Recently, it was suggested that this symporter can play critical roles in the immune response as well as in pregnancy and fetal growth.^19^ These findings appear to further support the idea that low adsorbable SGLT-1 inhibitors could represent a safer opportunity for controlling this symporter without affecting its functions in other tissues and organs.

As a prospective advancement in this research, solving the crystal structures of both hSGLT-1 and hSGLT-2 subtypes could provide significant progress useful to clarify additional functional aspects of these symporters and to support structure-based drug design of improved inhibitors. Moreover, it can be expected that the interest in this class of therapeutic agents and its future development will be significantly influenced by the results of ongoing clinical trials aimed to assess the cardiorenal effects of known gliflozins in diabetic and nondiabetic subjects.

## Abbreviations Used

CV: cardiovascular
DM: diabetes mellitus
EL5c: extracellular loop 5
GGM: glucose-galactose malabsorption
GLUT: sodium-independent facilitative glucose transporter
HbA1c: glycated hemoglobin
IGT: impaired glucose tolerance
IL: interleukin
SGLT: sodium-glucose cotransporter
STZ: streptozotocin
RT_G_: renal threshold for glucose excretion
TmG: tubular maximum glucose reabsorptive capacity
UGE: urinary glucose excretion
